# Machine learning-driven clinical decision support for liver cirrhosis: a gut microbiome-based web prediction model with explainable AI integration

**DOI:** 10.1186/s12876-026-04890-7

**Published:** 2026-05-06

**Authors:** Jianyuan Liu, Shiran He, Heng Zhang, Huanzhuo Mai, Xiaozhen Li, Hao Liang, Ping Cui, Liuyan Lan, Wenping Liao, Qianqian Huang, Huan Ning, Zhuoxin Li, Yunxiao Liang, Xing Yang, Jiegang Huang

**Affiliations:** 1 School of Public Health, Guangxi Medical University, Nanning, Guangxi China; 2 Guangxi Academy of Artificial Intelligence, Nanning, Guangxi China; 3 Guangxi Key Laboratory of AIDS Prevention and Treatment, Guangxi Medical University, Nanning, Guangxi China; 4 Key Laboratory of Prevention and Control of Highly Prevalent Diseases (Guangxi Medical University), Education Department of Guangxi Zhuang Autonomous Region, Nanning, Guangxi China; 5 School of Public Health, Capital Medical University, Beijing, China; 6 Outpatient Department, People’s Hospital of Guangxi Zhuang Autonomous Region and Guangxi Academy of Medical Sciences, Nanning, Guangxi China; 7 Life Sciences Institute, Guangxi Medical University, Nanning, Guangxi China; 8 Office of Hospital Quality and Safety Management Committee, People’s Hospital of Guangxi Zhuang Autonomous Region and Guangxi Academy of Medical Sciences, Nanning, Guangxi China; 9 Department of Gastroenterology, People’s Hospital of Guangxi Zhuang Autonomous Region and Guangxi Academy of Medical Sciences, Nanning, Guangxi China; 10 Research Center, People’s Hospital of Guangxi Zhuang Autonomous Region and Guangxi Academy of Medical Sciences, Nanning, Guangxi China

**Keywords:** Gut microbiota, 16S ribosomal RNA gene amplicon sequencing, Liver cirrhosis, Machine learning, Web-based deployment

## Abstract

**Background:**

Liver cirrhosis (LC) is a chronic liver disease with global prevalence. Current diagnostic methods for LC still face limitations in safety and accessibility. We aimed to develop an interpretable machine learning (ML) prediction model for LC using gut microbes and deploy it as a web-based clinical decision support tool.

**Methods:**

Data were retrieved from PubMed and BioProject databases. Bioinformatics re-analysis and discriminant analysis effect size (LEfSe) analysis was conducted to preliminarily identify key genera associated with LC. Further feature selection was performed using Least Absolute Shrinkage and Selection Operator (LASSO) regression. The independent datasets were combined to form an integrated dataset, which was then subjected to five-fold cross-validation and leave-one-dataset-out (LODO) analysis. Model performance was evaluated using metrics such as the area under the receiver operating characteristic curve (AUC), and the optimal model was selected. The decision mechanism of the optimal model was interpreted using SHapley Additive exPlanations (SHAP), and the model was deployed as a web application using the Streamlit framework.

**Results:**

We ultimately included 11 datasets related to LC. The genera *Veillonella*, *Lachnospira*, *Romboutsia*, *Akkermansia*, *Erysipelatoclostridium*, *Prevotella*, *UCG.005*, and *Streptococcus* were identified as key predictors distinguishing LC patients from healthy controls. The Random Forest (RF) model demonstrated the best predictive performance (AUC in five-fold cross-validation: 0.875, 95% CI: 0.823–0.905; AUC in LODO analysis: 0.793, 95% CI: 0.702–0.940) and was deployed as an online LC prediction tool.

**Conclusion:**

The interpretable RF model, along with its web-based implementation, has the potential to provide decision support for healthcare professionals and shows promise as a valuable auxiliary tool for LC screening and early clinical intervention.

**Supplementary Information:**

The online version contains supplementary material available at 10.1186/s12876-026-04890-7.

## Introduction

Liver cirrhosis (LC) is a chronic liver disease characterized by structural alterations in the liver induced by regenerative nodules and diffuse fibrosis [1]. It has a global prevalence, affecting at least 122.6 million people worldwide, and is associated with high morbidity and mortality, ranking as the 11th leading cause of death [2, 3]. Moreover, LC is the most significant risk factor for hepatocellular carcinoma (HCC), with HCC incidence increasing 2.79- to 45.00-fold in cirrhotic patients compared to non-cirrhotic individuals [4]. Therefore, early screening for LC is crucial for timely identification and reversal of etiological factors, as well as for preventing disease progression and complications; however, it remains a major challenge in the management of LC [2]. Currently, liver biopsy remains the gold standard for LC diagnosis [5]. However, its invasive nature, requirement for specialized technical expertise, high cost, and potential complications limit its widespread use in population screening and primary healthcare settings [6]. Non-invasive diagnostic alternatives, such as serum biomarkers, ultrasound elastography, and computed tomography (CT), have demonstrated diagnostic accuracy but also face similar limitations in scalability and broad applicability [5]. Therefore, developing a novel, non-invasive, accurate, simple, and cost-effective method for LC detection is urgently needed.

Studies have shown that the liver possesses a unique immune architecture and serves as the largest immune organ [7]. In patients with LC, the immune system participates in disease progression through specific mechanisms. Under conditions of LC, numerous cytokines exacerbate fibrosis through mechanisms such as inducing monocyte/macrophage infiltration into liver tissue and promoting the proliferation of hepatic stellate cells [7]. Additionally, in patients with LC, hepatocytes and intrahepatic immune cells lose their ability to balance the normal context-dependent dichotomous responses of immune tolerance versus immune activation [8]. As the largest microbial community in the human body, the gut microbiota and its metabolites play a pivotal role in host defense and maintaining immune homeostasis. Different gut bacterial species exert distinct regulatory effects on the development of various components of the immune system. For instance, segmented filamentous bacteria promote the development of homeostatic Treg cells in the gut, while specific clusters of Clostridia induce pro-inflammatory Treg cells [9, 10]. Therefore, we hypothesize that the onset and progression of LC may be associated with alterations in the gut microbiota. Numerous studies have confirmed an association between LC and alterations in the gut microbiota. LC itself is linked to profound changes in gut flora and impairments in various defense levels of the intestinal barrier. Moreover, severe intestinal barrier disruption in cirrhosis correlates with translocation of viable bacteria, bacterial infections, and disease progression [11, 12]. Some systematic reviews have analyzed the diversity indices of LC and differential microbiota, among other aspects [13, 14]. However, these systematic reviews face several limitations. Firstly, the analytical methods used in the included studies vary, which can affect the consistency of the findings. Secondly, the results of the studies are heterogeneous, and some inconsistencies are challenging to account for. Moreover, it is difficult to fully utilize the important information from these studies to conduct more in-depth analyses. Finally, the feasibility of using gut microbiota to predict LC remains unexplored in these studies.

Gut microbiota has emerged as a promising diagnostic tool for LC [15–17]. In recent years, machine learning (ML) algorithms have gained traction for their potential to enhance predictive accuracy [18]. Systematic reviews have shown that ML approaches based on gut microbiota can effectively predict LC, representing a leading non-invasive diagnostic method with high clinical utility [19]. However, current research on this topic suffers from several limitations [19–22], including insufficient statistical power from small-scale cohorts (typically < 100 cases/controls), geographic restrictions limiting training data diversity to specific regional populations within single nations without external validation across diverse demographics, etiological oversimplification through exclusive focus on cirrhosis from singular causes (e.g., alcoholic, nonalcoholic, or hepatitis B-related liver disease), narrow computational scope with only 1–6 machine learning algorithms employed for comparative modeling, and critical translational barriers arising from opaque “black-box” predictive architectures that hinder clinical interpretation. Most fundamentally, despite theoretical advancements, the deployment of web-based predictive tools implementing these models for predicting LC remains limited, which hinders their practical implementation and real-world clinical utility [19].

In light of these challenges, it becomes essential to collect and utilize the raw sequencing data from different studies to perform re-analysis and systematic integrated analyses. Therefore, we will next acquire all publicly available 16 S ribosomal RNA (16 S rRNA) gene amplicon sequencing data related to LC and gut microbiota, and then reanalyze them using rigorous bioinformatics methods. We will integrate these datasets from diverse geographic regions and etiologies to establish training and validation datasets. Next, based on the training set, we will employ 10 ML algorithms to construct predictive models and evaluate them using the validation set to identify the optimal model. Subsequently, SHAP analysis will be applied to interpret the optimal model. Finally, we will deploy this model as a web-based application. Our study aims to enhance the accessibility, interpretability, and clinical practicality of LC screening, providing healthcare professionals with a simple yet reliable auxiliary tool for LC detection.

## Methods

### Search strategy and selection criteria

We systematically identified relevant articles and datasets pertaining to LC and gut microbiota by conducting comprehensive searches in the National Center for Biotechnology Information (NCBI) databases, including PubMed [23] and BioProject [24], covering all available publications up to March 2026. Our search strategy was based on keywords, MeSH terms, and synonyms (Supplementary Table S1).

Studies were selected according to the following criteria: (1) inclusion of a LC group and a distinguishable control group; (2) sample type of fecal samples or rectal swabs; (3) sequencing method of 16 S rRNA gene amplicon sequencing with publicly available data; and (4) exclusion of studies involving randomized controlled trials, animal experiments, in vitro studies, reviews, meta-analyses, comments, letters, poster abstracts, and studies with fewer than three samples in either the case or control group.

Two independent reviewers screened titles, abstracts, and full texts. Disagreements were resolved through discussion or, if necessary, consultation with a third reviewer. No language restrictions were imposed; studies in any language were considered if they met the inclusion criteria. The study protocol has been registered in PROSPERO (ID: CRD420261331543).

### Processing of raw data

We downloaded Sequence Read Archive (SRA) files from the NCBI and converted them into raw FASTQ files using the SRA Toolkit [25]. These FASTQ files were then imported into Quantitative Insights Into Microbial Ecology version 2 (QIIME 2) for processing and bioinformatics analysis [26]. After removing primers from the raw sequences, we used the Divisive Amplicon Denoising Algorithm 2 (DADA2) plugin in QIIME 2 to trim primer sequences, remove chimeras, and filter low-quality reads with quality scores below 35, ensuring high-confidence reads and minimizing the impact of sequencing errors on downstream analyses [26, 27]. Detailed trimming parameters for each study are provided in Supplementary Table S2. Following DADA2 denoising to generate amplicon sequence variant (ASV) and a representative sequence table, we used the “q2-feature-table” plugin in QIIME 2 to filter out low-abundance features and low-quality samples based on the interquartile range (IQR) to enhance the quality of downstream statistical analysis. A phylogenetic tree was constructed using the “q2-phylogeny” plugin, and the species of each representative ASV were annotated using a pre-trained Naive Bayes classifier based on the SILVA 138 reference database (clustering similarity of 99%) [28].

### Data analysis

We imported the filtered feature table, phylogenetic tree, and taxonomy files into RStudio 2026.01.1 for analysis using R-4.5.2. The R package “microeco” [29] was used to eliminate ASV classified as “mitochondria” or “chloroplasts” and to create a microtable object for subsequent analysis. To mitigate the impact of sequencing depth on diversity measurements and enable fair comparisons among samples, all samples in the ASV abundance matrix were randomly resampled based on the lowest sequencing depth within the dataset of each individual study, and the ASV feature table was normalized [30]. Alpha diversity indices, including richness (Observed Species, Chao1, and ACE), diversity (Shannon, Simpson, InvSimpson, and Fisher), and phylogenetic diversity (PD), were calculated using the “vegan” package to assess the overall structure of the gut microbiota. Beta diversity was analyzed using principal coordinate analysis (PCoA) and non-metric multidimensional scaling (NMDS) based on the Bray-Curtis distance, followed by permutational multivariate analysis of variance (PERMANOVA) and analysis of similarities (ANOSIM) to evaluate similarities between groups. Additionally, Venn diagrams were constructed using the R package “VennDiagram” to display ASV intersections between different groups.

Differential microbial features between case and control groups in each study were identified using the linear discriminant analysis effect size (LEfSe) method (LDA score ≥ 2) via the R package “SpiecEasi” [31]. To stabilize variance, mitigate compositionality effects, and address comparability issues among features with different abundance levels, arcsine square root transformation and z-score normalization were applied to the microbiome relative abundance data before LEfSe analysis. The R package “Tax4Fun2 [32]” was used to predict Kyoto Encyclopedia of Genes and Genomes (KEGG) [33, 34] functional pathways associated with microbial communities based on 16 S rRNA gene sequencing data classified by the SILVA 99Ref database. We compared functional abundances between groups to identify significantly different functional pathways. Forest plots for comparing alpha diversity between case and control groups were generated using the R package “meta”. Heterogeneity was assessed using I^2^, with I^2^ > 45% indicating significant heterogeneity and prompting the use of a random-effects model; otherwise, a fixed-effects model was used. Results were evaluated using standardized mean differences (SMD) and corresponding 95% confidence intervals (CI).

### Key microbiota selection

The control group “healthy control (HC)” was assigned a value of “0”, and the case group “LC” was assigned a value of “1”, with group status as the dependent variable “LC”. Relative abundance data after arcsine square root transformation and z-score normalization from multiple datasets were first integrated into a single dataset (code for transformations available in Supplementary Table S3). Batch effects among datasets were then corrected using PLSDAbatch analysis [35]. A two-step approach was used for feature selection. First, LEfSe analysis was performed on the batch-effect-corrected data to identify differentially abundant genera. Subsequently, further screening of the variables selected in the first step was conducted using Least Absolute Shrinkage and Selection Operator (LASSO) regression analysis. This analysis was performed using the R package “glmnet”, with the optimal λ value determined via 10-fold cross-validation to optimize the model and select potential factors. Feature selection via LASSO was performed independently only on the training fold of each cross-validation iteration, adopting a nested cross-validation strategy (i.e., within each training fold, 10-fold cross-validation was used to select the optimal λ), without using any information from the test folds. Correlation analysis of the finally selected variables and visualization of the results via heatmaps were then performed. To evaluate the robustness of the selected microbial genera, we conducted a sensitivity analysis based on the leave-one-dataset-out (LODO) procedure: all datasets except one were used for training, and the held-out dataset was used for testing. During each LODO iteration, the LASSO selection based on the training set was performed de novo strictly within the training folds.

### Model development, evaluation, and interpretation

Using the above independent and dependent variable data, we comprehensively selected the 10 most common ML algorithms based on existing studies [19, 20, 36] to build predictive models: Logistic Regression (LR), Random Forest (RF), Extreme Gradient Boosting (XGB), Light Gradient Boosting Machine (LGBM), Adaptive Boosting (AdaBoost), K-Nearest Neighbors (KNN), Support Vector Machine (SVM), Gaussian Naive Bayes (GNB), Decision Tree (DT), and Multilayer Perceptron (MLP). First, five-fold cross-validation was performed on the integrated dataset for internal validation: the integrated dataset was randomly partitioned into five equal-sized folds. Each fold was used once as the validation set while the remaining four folds served as the training set, and this process was repeated five times. Subsequently, LODO analysis was conducted for external validation. It should be noted that for LODO analysis, the data partitioning was performed on the original dataset prior to batch effect correction. After partitioning, batch effects were corrected only on the training set using PLSDAbatch analysis, while the validation set remained uncorrected to avoid data leakage. We performed hyperparameter tuning for all machine learning models using grid search combined with five-fold cross-validation within each training fold to select the optimal hyperparameters. The hyperparameter grids used are documented in Supplementary Table S4.

Model performance was evaluated comprehensively based on discrimination, calibration, and clinical utility to select the best model. Discrimination was assessed using the area under the receiver operating characteristic curve (AUC) [37] and Matthews Correlation Coefficient (MCC, which considers true positive [TP], false positive [FP], true negative [TN], and false negative [FN], ranging from − 1 to 1, with 1 indicating perfect prediction, 0 random prediction, and − 1 inverse prediction) [38]. Calibration was evaluated using calibration curves and the Brier score (measuring prediction accuracy, ranging from 0 to 1, with lower values indicating higher accuracy) [39]. Clinical applicability was assessed via decision curve analysis (DCA) and mean Net Benefit over thresholds from 0.1 to 0.9 (NB-average) [40]. The SHAP framework was used to evaluate feature contributions to model predictions. To assess the robustness of model performance estimates, uncertainty measures were calculated for all evaluation metrics. For the AUC, 95% CIs were computed. In the five‑fold cross‑validation, DeLong’s method was applied to the pooled predictions from all five folds. In the LODO analysis, the 95% CI for AUC was derived using the empirical percentiles (2.5th–97.5th) of the ten AUC values obtained from the ten LODO iterations. For MCC, Brier score, and NB‑average, uncertainty was expressed as mean ± standard deviation (SD) across the five cross‑validation folds and ten LODO iterations, respectively.

All predictive models and SHAP analyses were implemented in Python 3.11, leveraging libraries such as “sklearn”, “xgboost”, “lightgbm”, “matplotlib”, “scipy”, and “shap”.

### Model deployment and web application

The optimal machine learning model, trained on the entire integrated dataset, was encapsulated using Python 3.11. For interactive deployment, the model was deployed as a web application using Streamlit [41], an open-source Python framework enabling dynamic data apps with minimal code, facilitating real-time user interaction through a publicly accessible interface.

## Results

### Study characteristics

**Fig. 1:**
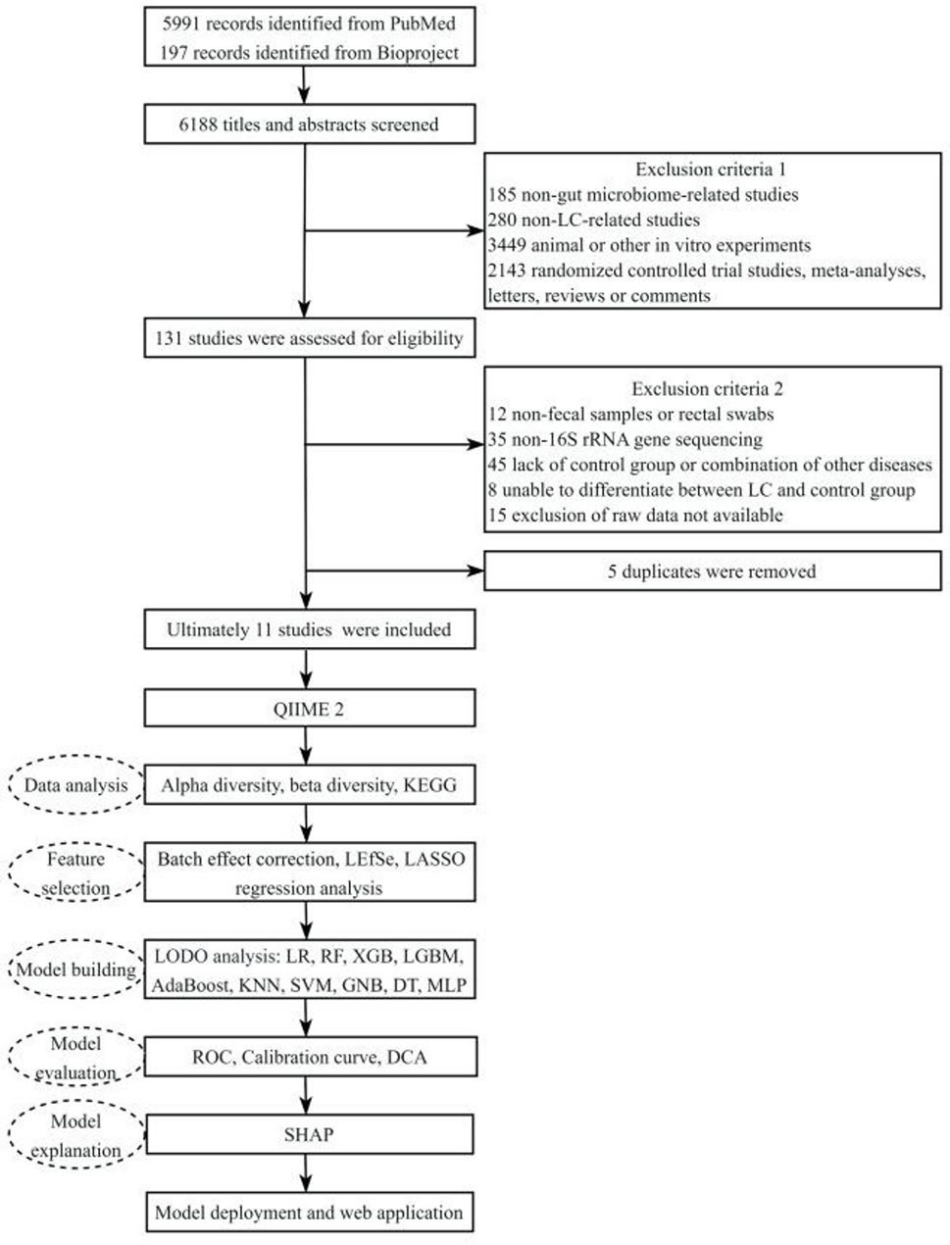
Flowchart of this study. *LC* liver cirrhosis, *QIIME 2* Quantitative Insights Into Microbial Ecology version 2, *LEfSe* linear discriminant analysis effect size, *KEGG* Kyoto Encyclopedia of Genes and Genomes, *LASSO* Least Absolute Shrinkage and Selection Operator, *LODO* leave-one-dataset-out, *LR* Logistic Regression, *RF* Random Forest, *XGB* Extreme Gradient Boosting, *LGBM* Light Gradient Boosting Machine, *AdaBoost* Adaptive Boosting, *KNN* K-Nearest Neighbors, *SVM* Support Vector Machine, *GNB* Gaussian Naive Bayes, *DT* Decision Tree, *MLP* Multilayer Perceptron, *ROC* Receiver operating characteristic, *DCA* Decision curve analysis, *SHAP* SHapley Additive exPlanations

According to the search criteria, we identified 5,991 studies from PubMed and 197 from BioProject, of which 11 studies met the inclusion criteria related to LC were included in the subsequent analysis (Shi et al., 2025 [42]; Sun et al., 2025 [43]; Gulyaeva et al., 2025 [44]; Li et al., 2022 [45]; Zhong et al., 2021 [46]; Zheng et al., 2020 [47]; Chen et al., 2020 [48]; Caussy et al., 2019 [49]; Liu et al., 2018 [50]; Iebba et al., 2018 [51]; NA, BioProject number: PRJEB32568) (Fig. 1). Among the 11 included studies, the causes of cirrhosis or fibrosis were specified as follows: 1 study on LC caused by alcoholic fatty liver [46], 1 study on LC caused by nonalcoholic fatty liver disease [49], and 4 studies on LC caused by chronic hepatitis B [42, 43, 45, 48]. The remaining studies did not specify the cause of cirrhosis. All control groups consisted of healthy controls and did not include patients with early-stage fibrosis. Detailed diagnostic methods and exclusion criteria are provided in Supplementary Table S5. These 11 studies were conducted in 5 countries, with 8 from China, 1 from the United States, 1 from Russia, and 1 from Italy. A total of 890 individual samples were included (LC, *n* = 427; healthy control (HC), *n* = 357; HCC, *n* = 106). More comprehensive details about the included studies are presented in Table 1.

**Table 1:** Study characteristics of the included studies

Author, Year [Ref]	PMID	BioProject accession number	Region, Country	Study period	16 S rRNA Variable Region, Sequencing Platform, Sequencing technology	Sample size
Shi et al., 2025 [42]	40458519	PRJNA1259947	Beijing, China	2022.12–2023.08	V3–V4, Illumina NovaSeq 6000, Paired-end sequencing	LC = 83, HC = 40
Sun et al., 2025 [43]	41122171	PRJNA784025	Beijing, China	2019.07–2021.08	V3–V4, Illumina NovaSeq, Paired-end sequencing	LC = 99, HC = 50
Gulyaeva et al., 2025 [44]	40069378	PRJNA1208993	Moscow, Russia	NA	V4, DNBSEQ-G50, Paired-end sequencing	LC = 30, HC = 22
Li et al., 2022 [45]	35733959	PRJNA838083	Guangdong, China	2020.10–2021.07	V4, Illumina NovaSeq 6000, Paired-end sequencing	LC = 24, HCC = 22, HC = 15
Zhong et al., 2021 [46]	33816353	PRJNA690835	Guangxi, China	NA	V3–V4, Illumina MiSeq, Paired-end sequencing	LC = 17, HC = 27
Zheng et al., 2020 [47]	32281295	PRJNA540574	Jilin, China	2017.03–2018.04	V4, Illumina HiSeq 2500, Paired-end sequencing	LC = 24, HCC = 75, HC = 20
Chen et al., 2020 [48]	32265857	PRJNA558158	Xiamen, China	2017.12–2018.05	V3–V4, Illumina HiSeq 2500, Paired-end sequencing	LC = 25, HC = 21
Caussy et al., 2019 [49]	30926798	PRJEB28350	California, America	2011.12– 2017.12	V4, Illumina MiSeq, Single read sequencing	LC = 26, HC = 117
Liu et al., 2018 [50]	29780327	PRJNA445763	Heilongjiang, China	2015.12–2016.12	V3–V4, Illumina MiSeq, Paired-end sequencing	LC = 36, HC = 20
Iebba et al., 2018 [51]	29844325	PRJNA471972	Rome, Italy	NA	V3–V4, Illumina MiSeq, Paired-end sequencing	LC = 52, HC = 20
NA	NA	PRJEB32568	Shandong, China	NA	NA, Illumina HiSeq 2500, Single read sequencing	LC = 11, HCC = 9, HC = 5

*QIIME 2* Quantitative Insights Into Microbial Ecology version 2, *NA* Not Available, *LC* liver cirrhosis, *HC* Healthy Control, *HCC* Hepatocellular carcinoma

### Gut microbial diversity significantly decreases with the onset and progression of LC

Compared to the HC group, the summary estimation of all alpha diversity indices in the LC group showed a significant decrease (SMD = -0.31; 95% CI: -0.41 to -0.21; *P* < 0.001; Supplementary Figure S1). Specifically, the ACE, Shannon, Simpson, and InvSimpson diversity metrics in the LC group were significantly downregulated (*P* < 0.05, Supplementary Figure S1). Other indices (Observed, Chao1, Fisher, PD) exhibited downward trends but did not reach statistical significance (Figure S1). Compared to the LC group, the summary estimation of all alpha diversity indices in the HCC group showed a downward trend but were not statistically significant (Supplementary Figure S2).

NMDS analysis using ANOSIM revealed significant differences in species composition between the HC and LC groups [43, 44, 48, 49, 51] and among the HC, LC, and HCC groups [45, 47, 24] (*P* < 0.05; Supplementary Figure S3). Similarly, PCoA analysis using PERMANOVA confirmed these compositional differences across the same group comparisons. (Supplementary Figure S4).

Venn diagrams illustrating ASV overlaps among groups indicated differences in gut microbial composition at the ASV level (Supplementary Figure S5).

KEGG pathway prediction based on 16 S rRNA gene sequences revealed that compared to HC, microbial functions such as Xenobiotics biodegradation and metabolism, Neurodegenerative diseases, Metabolism of terpenoids and polyketides, Metabolism of other amino acids, Lipid metabolism, Drug resistance: Antimicrobial, and Carbohydrate metabolism were significantly increased in LC (Supplementary Figure S6). No significant changes in microbial functions were observed between HCC and LC (Supplementary Figure S7).

### Key microbiota for LC prediction: *Veillonella*, *Lachnospira*, *Romboutsia*, *Akkermansia*, *Erysipelatoclostridium*, *Prevotella*, *UCG.005*, and *Streptococcus*

**Fig. 2:**
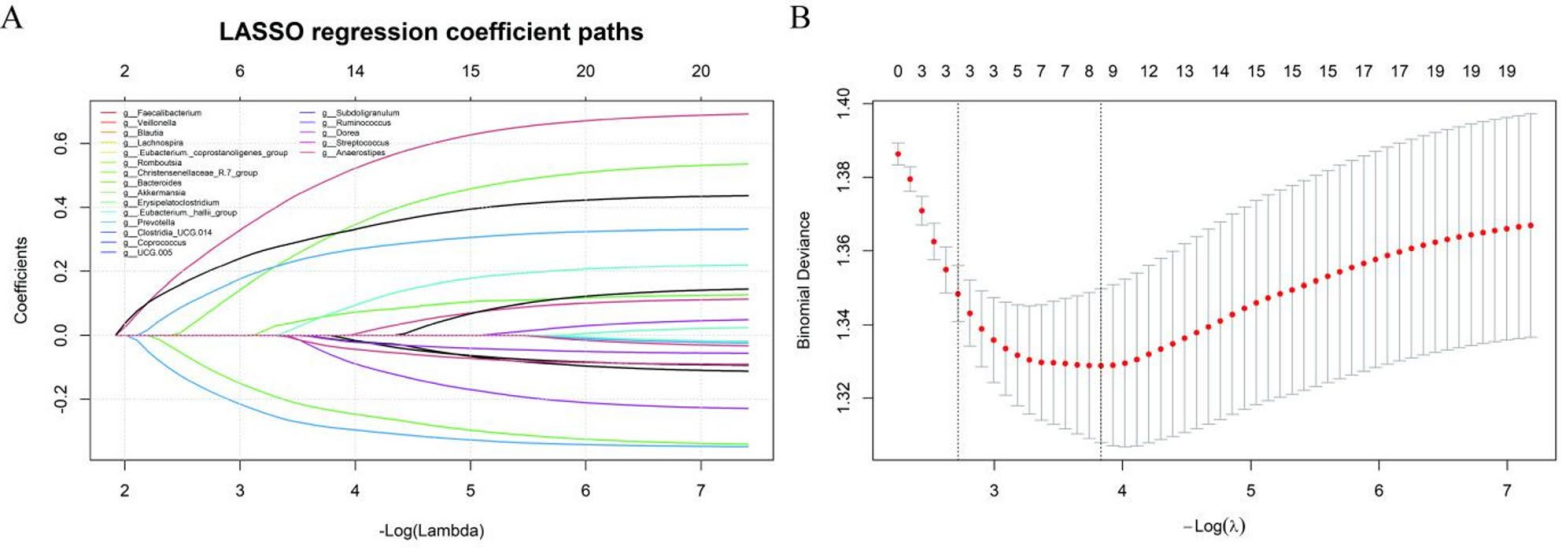
LASSO plots: **A** LASSO coefficient path plots for 20 variables. Each curve shows the coefficient path for each predictor. The vertical axis represents the coefficient value. The lower horizontal axis shows -log(λ), and the upper axis indicates the number of non-zero coefficients in the model at that λ. As log(λ) increases, the coefficients shrink toward zero. **B** Cross-validation curves of the LASSO model (10-fold cross-validation). The x-axis represents the negative log penalty coefficient (-log λ), and the y-axis shows the deviance, with smaller values indicating better model fit. Numbers above indicate the number of remaining variables at each λ. The right vertical dotted line marks λ-min, where the deviance is minimized, indicating the best fit. The left vertical dotted line marks λ-se, one standard error from the minimum deviance, where the model is simpler with fewer variables but still fits well

After batch effect correction, batch effects among datasets were substantially mitigated, while the biological differences between conditions were preserved (Supplementary Figure S8). Based on LEfSe analysis results (LDA ≥ 2 and *P* < 0.05), 20 genera were selected for further LASSO regression analysis. In the LASSO regression analysis, 8 variables were ultimately identified using the optimal λ value (λ-min): *Veillonella*, *Lachnospira*, *Romboutsia*, *Akkermansia*, *Erysipelatoclostridium*, *Prevotella*, *UCG.005*, and *Streptococcus* (Fig. 2). Correlation analysis showed weak correlations among the 8 variables (Supplementary Figure S9). In comparisons between the HCC and LC groups, based on LEfSe analysis results (LDA ≥ 2 and *P* < 0.05), 22 genera were selected, including *Clostridia_UCG-014*, *Agathobacter*, and *Turicibacter*; details are provided in Supplementary Figure S10.

**Fig. 3:**
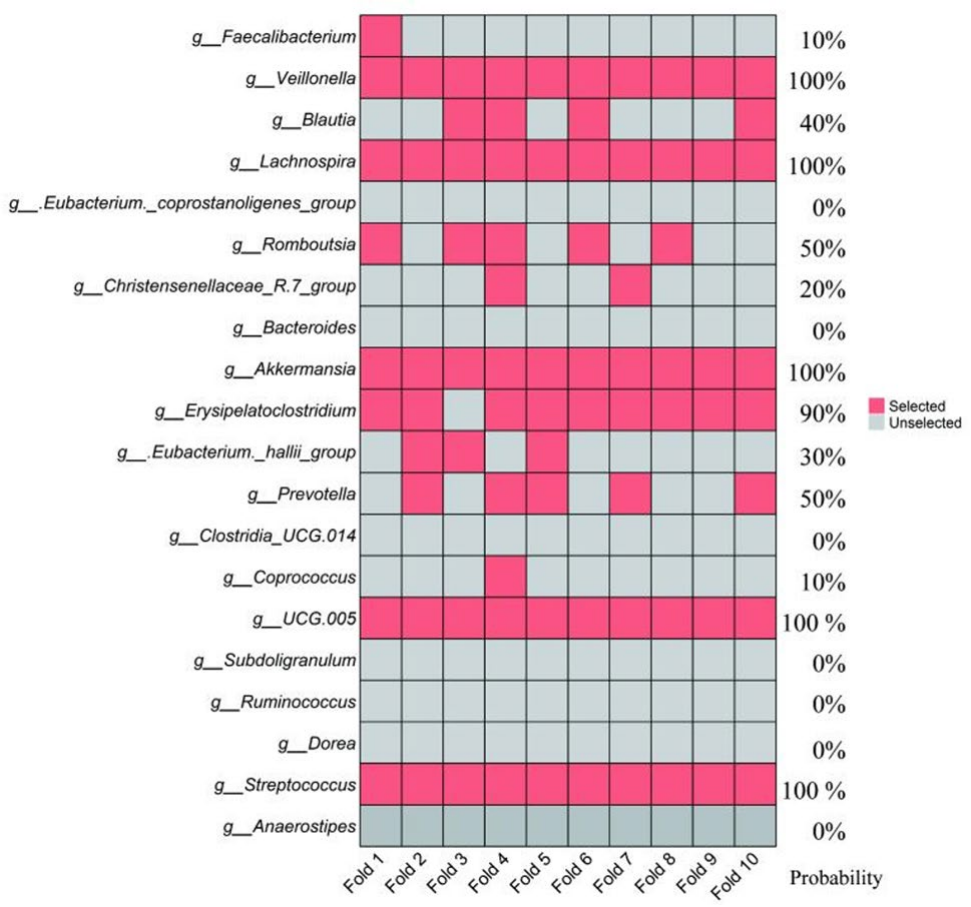
Sensitivity analysis of feature selection stability across LODO training folds. The heatmap illustrates the selection consistency of the 20 LEfSe-derived candidate genera during LASSO regression within each LODO training fold. Red cells indicate selection and white cells indicate non-selection. The eight genera retained in the final model exhibited selection frequencies ≥ 50% across all ten iterations, demonstrating high stability, whereas the remaining genera showed lower and more variable selection frequencies

Sensitivity analysis across the 10 LODO training folds confirmed the robustness of the selected features. The eight core genera (*Veillonella*, *Lachnospira*, *Romboutsia*, *Akkermansia*, *Erysipelatoclostridium*, *Prevotella*, *UCG.005*, and *Streptococcus*) were consistently selected by LASSO in a majority of the iterations (selection frequency ≥ 50%). In contrast, the other 12 candidate genera identified by LEfSe showed markedly lower selection frequencies (selection frequency < 50%) (Fig. 3).

### RF model: optimal discrimination, calibration, and clinical utility

Based on the availability of abundance data for the 8 selected genera, we formed a dataset comprising 10 studies for machine learning modeling (Table 2). Dataset PRJNA471972 was excluded because it lacked raw abundance data for some of the 8 selected genera.

**Table 2:** The sample size of various datasets used for machine learning modeling

Author, Year [Ref]	Sample size	Total
Shi et al., 2025 [42]	LC = 64, HC = 28	LC = 273, HC = 263
Sun et al., 2025 [43]	LC = 73, HC = 48	
Gulyaeva et al., 2025 [44]	LC = 21, HC = 18	
Zhong et al., 2021 [46]	LC = 10, HC = 20	
Zheng et al., 2020 [47]	LC = 19, HC = 13	
Chen et al., 2020 [48]	LC = 17, HC = 11	
Caussy et al., 2019 [49]	LC = 18, HC = 90	
Liu et al., 2018 [50]	LC = 25, HC = 17	
NA, BioProject: PRJEB32568 [24]	LC = 9, HC = 4	
Li et al., 2022 [45]	LC = 17, HC = 14	

*NA* Not Available, *LC* Liver cirrhosis, *HC* Healthy control

**Fig. 4:**
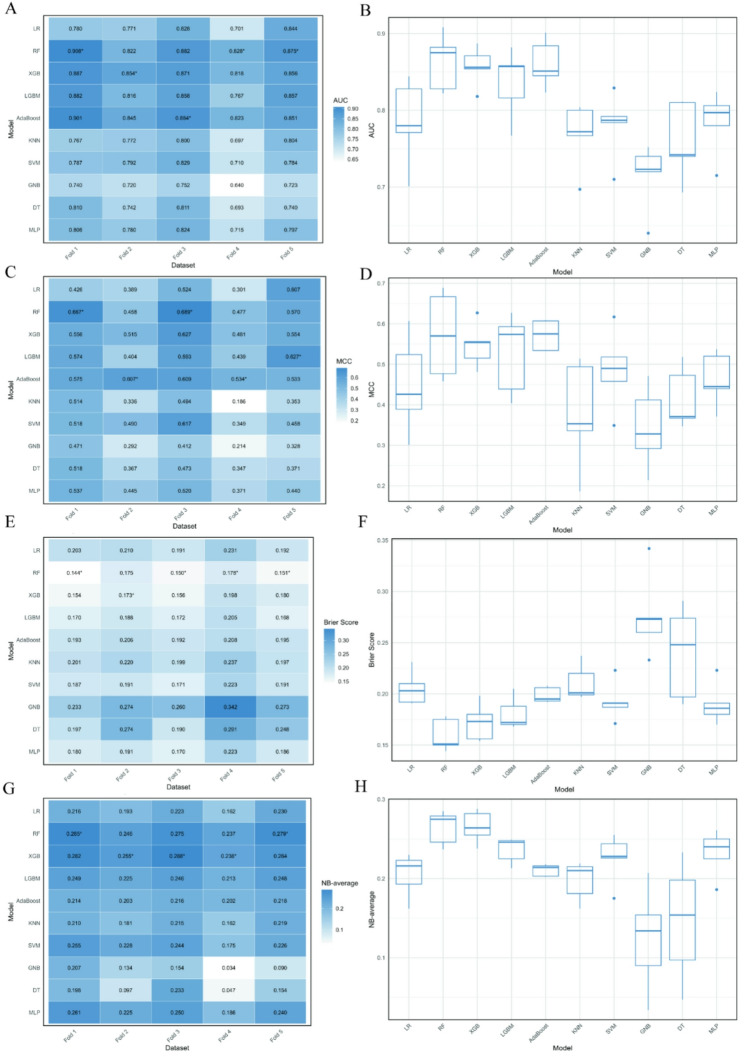
Evaluation of 10 machine learning models using five‑fold cross‑validation. **A** Heatmap of AUC; **B** Boxplot of AUC; **C** Heatmap of MCC; **D** Boxplot of MCC; **E** Heatmap of Brier score; **F** Boxplot of Brier score; **G** Heatmap of NB-average; **H** Boxplot of NB-average. In the five‑fold cross‑validation, the integrated dataset was randomly partitioned into five equal‑sized folds. Each fold was held out once as the validation set while the remaining four folds were used for training, and this process was repeated five times. This approach assesses model generalizability and stability. In the heatmaps (**A**, **C**, **E**, **G**), each column corresponds to one fold of the cross‑validation. For AUC, MCC, and NB-average, larger values indicate better performance, with the highest value in each fold marked by an asterisk (*). For the Brier score, smaller values indicate better performance, with the lowest value in each fold marked by an asterisk (*). The boxplots (**B**, **D**, **F**, **H**) show the distribution of AUC, MCC, Brier score, and NB-average values across the five folds for each model. The box represents the interquartile range (IQR), whiskers extend to the most extreme points within 1.5 × IQR, and outliers are shown as individual points. MCC Matthews Correlation Coefficient, NB-average mean Net Benefit over thresholds from 0.1 to 0.9

**Fig. 5:**
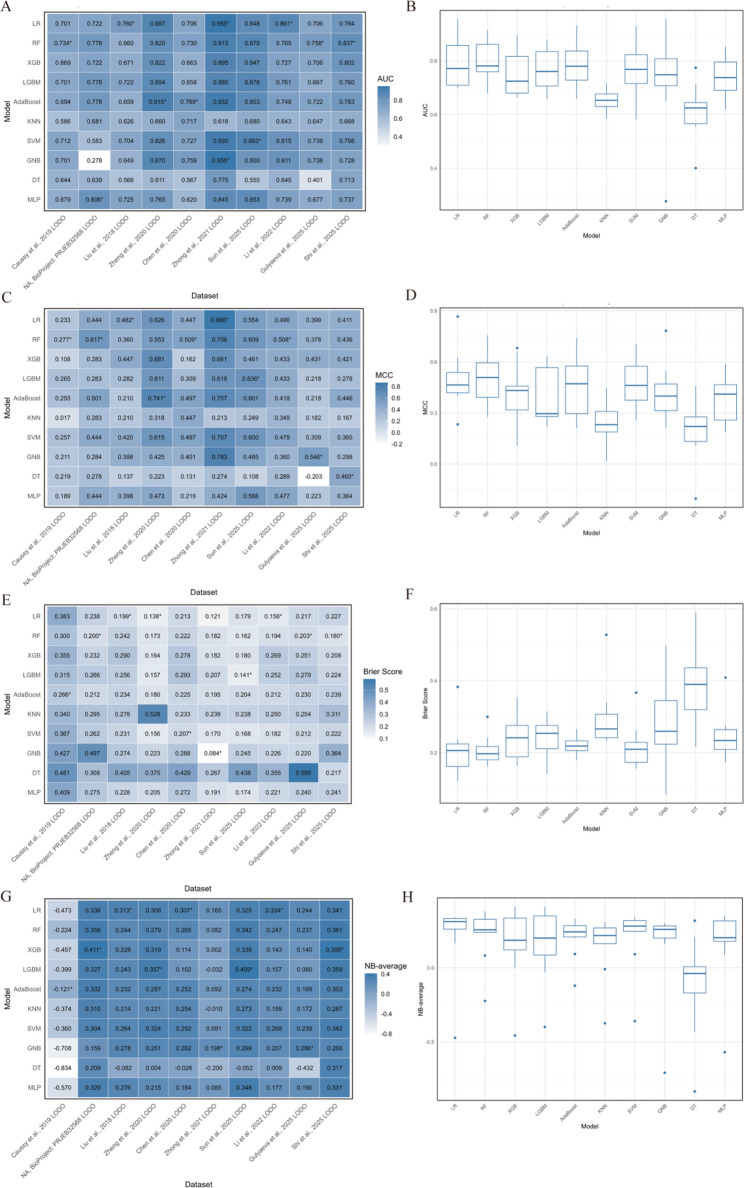
Evaluation of 10 machine learning models in the LODO analysis. **A** Heatmap of AUC; **B** Boxplot of AUC; **C** Heatmap of MCC; **D** Boxplot of MCC; **E** Heatmap of Brier score; **F** Boxplot of Brier score; **G** Heatmap of NB-average; **H** Boxplot of NB-average. In the LODO analysis, all datasets except one were used for training, and the held-out dataset was used for testing to assess model generalizability. In the heatmaps (**A**, **C**, **E**, **G**), for AUC, MCC, and NB-average, larger values indicate better performance, with the highest value in each dataset marked by an asterisk (*). For the Brier score, smaller values indicate better performance, with the lowest value in each dataset marked by an asterisk (*). The boxplots (**B**, **D**, **F**, **H**) show the distribution of AUC, MCC, Brier score, and NB-average values across datasets for each model. The box represents the interquartile range (IQR), whiskers extend to the most extreme points within 1.5 × IQR, and outliers are shown as individual points. *LODO* leave-one-dataset-out, *MCC* Matthews Correlation Coefficient, *NB-average* mean Net Benefit over thresholds from 0.1 to 0.9

Figure 4 and Figure S11 present the performance of 10 machine learning models evaluated by the AUC metric across five-fold cross-validation. In Fig. 4A, the RF model consistently achieved the highest AUC values in most folds (0.908, 0.828, and 0.875, respectively, in folds 1, 4, and 5, Fig. 4A, Figure S11A), while XGB and Asaboost also performed strongly in fold 2 and fold 3 (0.854 and 0.884, Fig. 4A, Figure S11A). Figure 4B further reveals that RF attained the highest median AUC (Fig. 4B) with a narrow interquartile range, indicating both superior performance and robustness. XGB and LGBM also demonstrated high medians and compact distributions, whereas GNB and DT had the lowest medians and wider spreads, reflecting inconsistent performance (Fig. 4B). Figure 5 and Figure S12 present the performance of 10 machine learning models evaluated using AUC metric in the LODO analysis. Figure 5A displays the AUC values for each model across the different held-out datasets. Notably, RF achieved the highest AUC values in several folds, while LR also demonstrated strong performance (Fig. 5A). Figure 5B summarizes the statistical distribution of AUC scores across all held-out datasets for each model. RF exhibited the highest median AUC, followed by LR, AdaBoost, and SVM (Fig. 5B). KNN and DT had the lowest medians (Fig. 5B).

Figure 4 presents the performance of 10 machine learning models evaluated using the MCC metric across five-fold cross-validation. Figure 4C displays the MCC values for each model across the five folds. The RF model achieved the highest MCC values in most folds, with notable scores of 0.667 and 0.689 in folds 1 and 3, respectively (Fig. 4C). Figure 4D summarizes the statistical distribution of MCC scores across all folds for each model. RF exhibited the highest median MCC (Fig. 4D), indicating both superior performance. Figure 5 presents the performance of 10 machine learning models evaluated using the MCC metric in the LODO analysis. Figure 5C displays the MCC values for each model across the different held-out datasets. Notably, RF achieved the highest MCC values in most folds (Fig. 5C). Figure 5D summarizes the statistical distribution of MCC scores across all held-out datasets for each model. RF exhibited the highest median MCC with a relatively narrow interquartile range (Fig. 5D).

Figure 4 and Figure S11 present the performance of 10 machine learning models evaluated using the Brier score across five-fold cross-validation. The Brier score measures the calibration of probabilistic predictions, with lower values indicating better performance. Figure 4E and Figure S11B displays the Brier scores for each model across the five folds. The RF model consistently achieved the lowest Brier scores in most folds, with notable values of 0.144, 0.150, 0.178, and 0.151 in folds 1, 3, 4, and 5, respectively (Fig. 4E). Figure 4F summarizes the statistical distribution of Brier scores across all folds for each model. RF had the lowest median Brier score with a narrow interquartile range, reflecting excellent and stable calibration (Fig. 4F). XGB and LGBM also showed low medians and compact distributions. Conversely, GNB and DT had the highest medians and the widest spreads (Fig. 4F). Figure 5 and Figure S13 present the performance of 10 machine learning models evaluated using the Brier score in the LODO analysis. Figure 5E and Figure S13 displays the Brier scores for each model across 10 held-out datasets. The LR and RF models achieved the lowest Brier scores in most folds. Figure 5F summarizes the statistical distribution of Brier scores across all held-out datasets for each model. RF had the lowest overall Brier score (Fig. 5F), reflecting superior and stable calibration. DT had the highest score, confirming its poor calibration (Fig. 5F).

Figure 4 and Figure S11 present the performance of ten machine learning models evaluated using the average Net Benefit metric across five-fold cross-validation. Net Benefit, derived from decision curve analysis, quantifies the clinical utility of models, with higher positive values indicating greater benefit. Figure 4G displays the average Net Benefit for each model across the five folds. The RF model achieved the highest Net Benefit values in folds 1 and 5, with scores of 0.285 and 0.279, respectively (Fig. 4G). XGB also demonstrated strong clinical utility, particularly in folds 2, 3, and 4 (0.255, 0.288, and 0.238, respectively, Fig. 4G). Figure 4H summarizes the statistical distribution of Net Benefit scores across all folds for each model. RF exhibited the highest median Net Benefit, followed closely by XGB and LGBM ( Fig. 4H). GNB and DT had the lowest medians (Fig. 4H). Figure 5 and Figure S14 present the performance of 10 machine learning models evaluated using the average Net Benefit metric in the LODO analysis. Figure 5G displays the average Net Benefit for each model across 10 held-out datasets. LR demonstrated strong clinical utility in multiple datasets (Fig. 5G). In contrast, DT and GNB consistently yielded the lowest Net Benefit values across most datasets. Figure 5H summarizes the statistical distribution of Net Benefit scores across all held-out datasets for each model. LR exhibited the highest median Net Benefit, followed by RF and SVM (Fig. 5H). However, DT also showed the widest range, with extreme negative values, reflecting instability

To further quantify model robustness and facilitate comparison across algorithms, we summarized the performance of all ten models with associated uncertainty estimates (Table 3). In the five‑fold cross‑validation, the RF model achieved a mean AUC of 0.875 (95% CI: 0.823–0.905), an MCC of 0.572 ± 0.106, a Brier score of 0.160 ± 0.016, and an NB‑average of 0.264 ± 0.021. In the LODO external validation, RF maintained robust performance with a median AUC of 0.793 (95% CI: 0.702–0.940), an MCC of 0.500 ± 0.142, a Brier score of 0.206 ± 0.041, and an NB‑average of 0.221 ± 0.177. Among the remaining models, LR and XGB also demonstrated competitive performance, whereas KNN, GNB, and DT exhibited lower accuracy and greater variability.

**Table 3:** Performance metrics with uncertainty estimates for all ten machine learning models

Model	Validation	AUC (95% CI)	MCC (mean ± SD)	Brier score (mean ± SD)	NB-average (mean ± SD)
LR	Five-fold CV	0.780 (0.708–0.842)	0.449 ± 0.119	0.205 ± 0.016	0.205 ± 0.028
	LODO	0.787 (0.676–0.905)	0.495 ± 0.166	0.207 ± 0.073	0.220 ± 0.250
RF	Five-fold CV	0.875 (0.823–0.905)	0.572 ± 0.106	0.160 ± 0.016	0.264 ± 0.021
	LODO	0.793 (0.702–0.940)	0.500 ± 0.142	0.206 ± 0.041	0.221 ± 0.177
XGB	Five-fold CV	0.856 (0.822–0.885)	0.547 ± 0.055	0.172 ± 0.018	0.265 ± 0.020
	LODO	0.752 (0.664–0.884)	0.409 ± 0.186	0.241 ± 0.060	0.164 ± 0.256
LGBM	Five-fold CV	0.857 (0.772–0.880)	0.527 ± 0.099	0.181 ± 0.016	0.236 ± 0.016
	LODO	0.766 (0.660–0.879)	0.393 ± 0.167	0.239 ± 0.057	0.160 ± 0.244
AdaBoost	Five-fold CV	0.851 (0.825–0.899)	0.572 ± 0.037	0.199 ± 0.008	0.211 ± 0.008
	LODO	0.787 (0.667–0.928)	0.459 ± 0.191	0.220 ± 0.025	0.208 ± 0.133
KNN	Five-fold CV	0.772 (0.704–0.804)	0.377 ± 0.133	0.211 ± 0.017	0.197 ± 0.025
	LODO	0.653 (0.593–0.709)	0.243 ± 0.117	0.297 ± 0.089	0.151 ± 0.206
SVM	Five-fold CV	0.787 (0.717–0.825)	0.486 ± 0.097	0.193 ± 0.019	0.226 ± 0.031
	LODO	0.773 (0.610–0.922)	0.469 ± 0.142	0.218 ± 0.062	0.209 ± 0.212
GNB	Five-fold CV	0.723 (0.648–0.751)	0.343 ± 0.101	0.276 ± 0.040	0.124 ± 0.065
	LODO	0.729 (0.361–0.936)	0.419 ± 0.161	0.285 ± 0.118	0.152 ± 0.305
DT	Five-fold CV	0.742 (0.698–0.811)	0.415 ± 0.076	0.240 ± 0.045	0.146 ± 0.075
	LODO	0.612 (0.436–0.761)	0.192 ± 0.172	0.386 ± 0.108	-0.109 ± 0.326
MLP	Five-fold CV	0.797 (0.722–0.822)	0.463 ± 0.067	0.190 ± 0.020	0.232 ± 0.029
	LODO	0.745 (0.633–0.851)	0.380 ± 0.131	0.246 ± 0.066	0.157 ± 0.269

*Abbreviations*: *LR* Logistic Regression, *RF* Random Forest, *XGB* Extreme Gradient Boosting, *LGBM* Light Gradient Boosting Machine, *AdaBoost* Adaptive Boosting, *SVM* Support Vector Machine, *KNN* K-Nearest Neighbors, *GNB* Gaussian Naive Bayes, *DT* Decision Tree, *MLP* Multilayer Perceptron, *CV* Cross-validation, *LODO* Leave-one-dataset-out, *AUC* Area under the receiver operating characteristic curve, *CI* Confidence interval, *MCC* Matthews Correlation Coefficient, *SD* Standard deviation, *NB-average* Mean Net Benefit over thresholds 0.1–0.9

### SHAP identifies the contribution of key genera in LC prediction

**Fig. 6:**
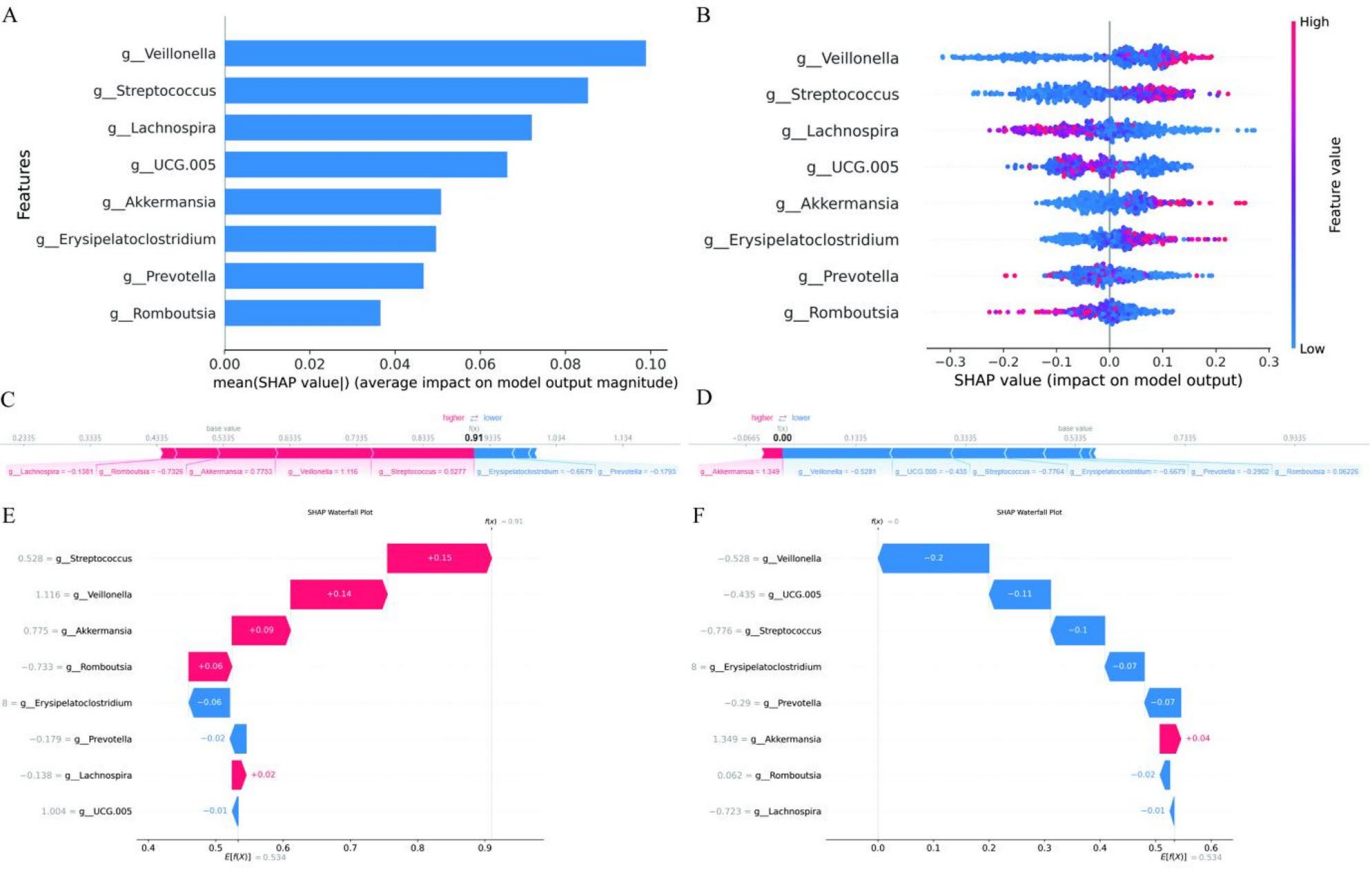
SHAP plots for RF in external validation set: **A** SHAP importance ranking plot of variables; **B** SHAP beeswarm plot (Each point represents an instance; red indicates higher feature values, blue indicates lower values. Features are ranked top to bottom by average SHAP value. Variables to the right of the SHAP value 0 line in red positively contribute, while those in blue negatively contribute); **C** / **D** SHAP force plot of one LC / HC subject in training set (Features are red/blue-coded to show their impact on increasing/decreasing the score, with those near the decision boundary having larger bars indicating greater impact); **E** / **F** SHAP waterfall plot of one LC / HC subject in the training set (The X-axis denotes the expected classifier output value. Each row shows each feature’s contribution relative to this expectation. Red/blue signifies positive/negative shifts. Features on the Y-axis have gray parameter readings)

Figure 6A ranks the 8 bacterial genera by their average magnitude of impact on the model’s predictive output, with *Veillonella* having the most significant effect, surpassing other features. The remaining genera, in descending order of impact, were *Streptococcus*,* Lachnospira*,* UCG.005*,* Akkermansia*,* Erysipelatoclostridium*,* Prevotella*,* and Romboutsia*. The SHAP beeswarm plot in Fig. 6B revealed the magnitude and direction of the impact of 8 dominant bacterial genera on the model’s predictive output. Among these, *Veillonella* and *Streptococcus* predominantly exhibited positive SHAP values, indicating that higher relative abundances of these genera were associated with increased model output (i.e., a push toward the “High” feature value class). In contrast, *Lachnospira* showed a clear trend of negative SHAP values, suggesting that elevated levels of this genus correlated with reduced model output (i.e., a push toward the “Low” feature value class). Genera including *UCG.005*, *Akkermansia*, *Erysipelatoclostridium*, *Prevotella*, and *Romboutsia* displayed mixed effects, with both positive and negative SHAP values observed across samples, reflecting context-dependent influences on the model prediction.

Force plots in Figs. 5D and 6C respectively explain individual predictions for one LC and one HC subject in the dataset. For the LC subject, *Lachnospira*, *Romboutsia*, *Akkermansia*, *Veillonella*, and *Streptococcus* positively influenced the prediction of LC, while *Erysipelatoclostridium* and *Prevotella* had negative impacts (Fig. 6C). The final prediction value (0.91) exceeded the base value (0.5335), leading to an LC prediction. For the HC subject, negative impacts predominated, resulting in an HC prediction (Fig. 6D).

SHAP waterfall plots in Figs. 5F and 6E correspond to the same two subjects mentioned above, respectively. For the LC subject, *Streptococcus* was the top positive contributor, followed by *Veillonella*, while *Erysipelatoclostridium* was the top negative contributor, followed by *Prevotella* (Fig. 6E). The model output (0.91) exceeded the baseline (0.534), confirming an LC prediction. For the HC subject, negative impacts predominated, resulting in an HC prediction (Fig. 6F).

### Web application for LC prediction

**Fig. 7:**
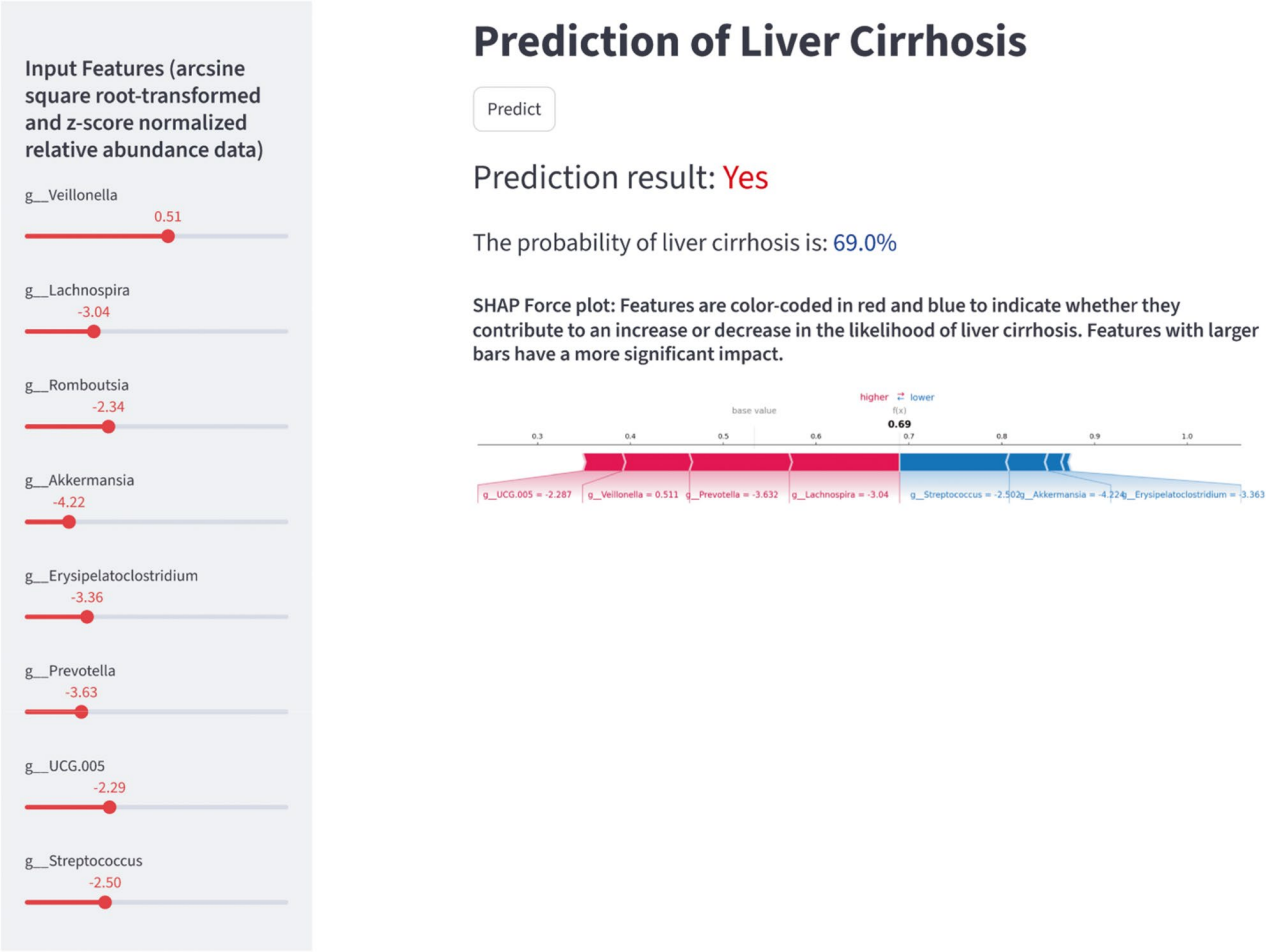
The online web-based application for predicting liver cirrhosis. Slide the slider on the left side of the page to adjust the numerical values of the features, then click the “Predict” button on the right side of the page to view the prediction results

The RF model developed from the integrated dataset has been successfully deployed on Streamlit for predicting LC in individuals. The left side of the page allows input of feature values (arcsine square root-transformed and z-score normalized relative abundance data), while the right side displays prediction results (Fig. 7). The web application is publicly accessible and permanently free to use [52].

## Discussion

Our study has several strengths: Firstly, by leveraging multinational and multiregional research datasets, we enhance the generalizability of the findings to some extent while potentially enabling reliable extrapolation to broader populations. This is further strengthened through the inclusive analysis of cirrhosis arising from diverse etiological pathways to ensure clinical relevance across heterogeneous patient groups. Secondly, we conducted a comprehensive comparison using 10 ML algorithms, with multidimensional model performance evaluation ensuring optimal model selection, and the analytical depth is augmented by SHAP-driven variable importance quantification, which provides clinically interpretable insights into feature contributions. Most importantly, the optimal model was deployed on a web-based platform via a freely accessible LC prediction web application. With only 8 variables, this stable model is more acceptable and scalable, and shows promise as an auxiliary tool to facilitate early screening and clinical diagnosis.

Alpha diversity encompasses the richness, evenness, or both of ecological communities [53]. Our results indicate that gut microbiota alpha diversity is lower in patients with LC than in healthy individuals, consistent with previous studies [13, 14]. This aligns with the general assumption that higher microbial diversity is beneficial for host health [54]. Compared to LC patients, alpha diversity indices in HCC patients showed a downward trend but were not significant. Existing studies show no consistency in alpha diversity changes in the gut microbiota of HCC patients [13]. However, given the high heterogeneity observed for some diversity indices, the pooled estimates should be regarded as indicative of an overall trend rather than a precise effect size. For beta diversity, PCoA and NMDS analyses revealed significant differences in species composition between HC and LC groups in at least 4 datasets and among HC, LC, and HCC groups in at least 2 datasets. This indicates that gut microbiota composition changes significantly with the onset and progression of LC, consistent with prior studies [13]. We employed rarefaction to normalize amplicon sequencing data, ensuring that within each dataset, diversity analyses were not systematically confounded by variation in sequencing depth. As demonstrated by Schloss, rarefaction offers distinct advantages in controlling for sequencing depth bias in both alpha and beta diversity analyses [30]. However, rarefaction may entail a loss of statistical power, and the compositional nature of microbiome data warrants additional caution in differential abundance analyses. Future studies can explore selecting the most appropriate strategies for different analytical objectives: for example, using rarefaction for diversity analyses while employing compositional data-specific methods for differential abundance analyses to balance result accuracy and statistical power.

The two-step filtering method used in this study, which performs preliminary screening by LEfSe followed by LASSO, may increase selection bias. However, it optimizes model construction by reducing the input features, alleviates the curse of dimensionality in high-dimensional data, and retains relevant and non-redundant features. Consequently, it improves the pattern recognition ability and prediction accuracy of the algorithm, reduces the risk of overfitting, and enhances the generalization ability [55]. We have performed an additional sensitivity analysis based on the LODO framework. The analysis shows that the eight genera reported in the text were selected by LASSO in ≥ 50% of the LODO iterations, whereas the other 12 candidate genera exhibited much lower selection frequencies. This analysis confirms that the final feature set is robust to variations in training cohort composition. Furthermore, the simplified feature subset improves model interpretability and helps identify key biomarkers in biomedical and other fields [56]. We performed feature selection on the integrated dataset with the goal of leveraging the advantages of data integration to identify more robust target microbial genera. However, this process may introduce optimistic bias into the subsequent cross-validation. To minimize such bias as much as possible, in the LODO analysis, we reintegrated, preprocessed, and corrected batch effects for the datasets within each training fold.

Based on LEfSe analysis, 20 differentially abundant genera were selected for further analysis. Subsequent LASSO regression set narrowed this down to 8 genera: *Veillonella*, *Lachnospira*, *Romboutsia*, *Akkermansia*, *Erysipelatoclostridium*, *Prevotella*, *UCG.005*, and *Streptococcus*. These 8 genera better distinguished LC and HC subjects than the other 12 genera. *Veillonella* is significantly increased in LC patients and may exacerbate the condition by activating inflammation and affecting gut barrier function [13, 53, 57]. In patients with LC, the abundance of *Lachnospira* is significantly reduced, and this reduction is strongly associated with the severity of the disease (e.g., MELD score) as well as specific metabolic alterations (such as decreased levels of tocopherol and 21-hydroxypregnenolone) [42]. In patients with LC and portal vein thrombosis, the abundance of *Romboutsia* in portal venous blood is significantly negatively correlated with the Child-Pugh score and MELD score, suggesting that this bacterium may be associated with the protection of liver function [58]. In patients with LC, the abundance of *Akkermansia* is significantly reduced. Additionally, in a mouse model of cirrhosis, supplementation with *Akkermansia muciniphila* markedly alleviated the area of liver fibrosis and reduced blood ammonia levels, suggesting that it may mitigate the progression of cirrhosis by maintaining the intestinal barrier and reducing endotoxemia [59]. *Erysipelatoclostridium* may be a protective factor in liver cirrhosis; however, elevated plasma nitrite levels (reflecting endothelial dysfunction) are associated with increased abundance of the genus *Erysipelatoclostridium* [60, 61]. *Prevotella* may play either a potential pathogenic role in the development and progression of liver cirrhosis or a protective role [62, 63]. This suggests that the effect of the genus Prevotella in liver cirrhosis may be strain-specific and co-regulated by multiple factors. No studies on UCG.005 and LC were found; thus, our finding may represent a novel observation. However, this is a statistical association derived from secondary analysis without experimental validation, and given the multiple testing inherent in microbiome studies, false-positive findings are possible. Mechanistic studies are needed to confirm this relationship. Systematic reviews indicate increased *Streptococcus* in LC patients [13, 53]. In summary, our findings confirm the associations of *Veillonella*, *Lachnospira*, *Romboutsia*, *Akkermansia*, *Erysipelatoclostridium*, *Prevotella*, and *Streptococcus* with LC, as reported in previous studies. Additionally, we may have made a novel discovery: *UCG.005* may have a significant relationship with LC.

KEGG analysis revealed significantly increased microbial functions like Xenobiotics biodegradation and metabolism, Metabolism of other amino acids, and Lipid metabolism in LC. Xenobiotics biodegradation and metabolism involves host-microbe interactions. LC patients, with impaired liver function, may fail to metabolize xenobiotics effectively, leading to their accumulation and increased liver burden [64]. Metabolism of other amino acids relates to amino acid metabolic disorders in LC patients, linked to reduced liver function, increased peripheral protein catabolism, and decreased amino acid clearance [65]. Lipid metabolism abnormalities in LC patients include impaired fatty acid synthesis/degradation and triglyceride accumulation [66]. However, functional predictions using Tax4Fun2 are based on 16 S rRNA gene sequences and rely on reference genomes; therefore, they represent inferred metagenomic content rather than direct measurements. These results should be considered hypothesis-generating and require validation through shotgun metagenomics or metabolomics.

In the five-fold cross-validation and LODO analysis, we comprehensively compared various models. The RF model demonstrated the best overall performance in terms of discrimination, calibration, and clinical utility. RF, with superior overall performance, was chosen as the best model for LC prediction. RF, an ensemble learning-based algorithm, enhances generalization and robustness by combining multiple decision trees, making it suitable for high-dimensional data and complex relationships [67]. In the RF model, 8 genera—*Veillonella*, *Lachnospira*, *Romboutsia*, *Akkermansia*, *Erysipelatoclostridium*, *Prevotella*, *UCG.005*, and *Streptococcus*—were key input features. SHAP explained the RF model’s individual subject predictions, clarifying its decision mechanism. This enhances model interpretability and credibility, aiding healthcare providers in evaluating diagnostic results and improving LC assessment quality and clinical utility.

We deployed the RF model on Streamlit, enabling user interaction via a web interface. Users can input feature data on the left and view predictions on the right, with SHAP force plots visualizing feature contributions. This visual personalized prediction model offers a simple and intuitive LC detection tool for healthcare professionals, holding new promise for LC screening and early clinical intervention, and enhancing the model’s translational value in real clinical settings. The deployed model is fully consistent with the RF model built on the integrated dataset described in this study and is locked to the analyses presented. This web application for predicting LC is currently free of charge and publicly accessible. It is worth noting that, due to the specialized nature of the tool, its current target users are healthcare professionals who have received relevant training in its use.

Although this study developed and validated a gut microbiota-based model for diagnosing LC and deployed a user-friendly online tool, several challenges remain on the path toward its widespread clinical application. First, fecal 16 S sequencing is not yet a routine clinical test; its implementation requires specialized laboratory equipment, bioinformatics pipelines, and trained personnel, which may be difficult to implement in resource-limited healthcare settings. Furthermore, the requirement for transformed microbiome data as input limits its practical clinical applicability. Widespread clinical adoption will require its integration into routine clinical workflows, the automation of standardization procedures, and validation in prospective studies. Second, the current cost of fecal 16 S sequencing may be higher than that of non-invasive tests such as the Fibrosis-4 (FIB-4) index, Enhanced Liver Fibrosis (ELF) test, and Vibration Controlled Transient Elastography (VCTE) [68–70]. Therefore, our microbiome model may not currently offer a cost advantage. Its potential clinical value could lie in providing unique diagnostic information or patient management insights beyond existing methods—for example, as a completely non-invasive approach, it may be suitable in specific scenarios such as when patients refuse blood draws, when imaging is unavailable or yields indeterminate results, or as a supplementary tool that offers new perspectives on disease mechanisms. Future rigorous health economic studies are needed to evaluate its cost-effectiveness within specific clinical pathways. Although it is not immediately applicable in clinical practice at the present stage, this approach can still serve as a valuable validation tool for researchers. Moreover, rigorously screened and reliable candidate microbial taxa and biomarkers may lay a solid foundation for future mechanistic investigations and clinical translation.

However, this study also has some limitations. Firstly, as a retrospective observational study, limiting causal inferences between dysbiosis and LC risk. Secondly, the sample size of this study may still be insufficient, which could limit the robustness of the model and pose a risk of overfitting. Therefore, future studies need to continue collecting and integrating data from various studies to further expand the sample size and validate the model in larger, multi-center prospective cohorts. Thirdly, as this study is a secondary analysis based on public high-throughput sequencing data, we did not have access to the original patients’ blood parameters to calculate FIB-4 scores or to ELF/VCTE results for direct comparison. Future studies are needed to compare this model with standard non-invasive methods such as FIB-4 and VCTE within the same cohort to determine its relative performance and additional clinical value. Fourthly, we integrated data from multiple countries and included various etiologies. Although a unified analytical pipeline, data normalization, and batch effects correction were applied to reduce heterogeneity and bias when integrating multi-source data, the effects might remain limited. Confounding stemming from geographical and etiological heterogeneity may still influence the observed microbial associations and limit the generalizability of the model to specific populations or cirrhosis subtypes. In addition, constrained by the number of included studies and the nature of microbiome data, this study did not conduct subgroup analyses. Future studies should seek validation in larger and more uniformly designed datasets. Finally, due to limitations in the currently available data, focusing solely on differences between healthy controls and liver cirrhosis may limit the model’s applicability, as it may be difficult to identify transitional stages such as early fibrosis and compensated cirrhosis. Therefore, at this stage, our model is likely closer to a “proof-of-concept classifier” demonstrating the feasibility of using gut microbiota for LC detection, rather than a ready-to-use diagnostic tool intended to replace existing diagnostic methods. However, it may serve as a complement or supplement to current approaches. Future studies should incorporate a more comprehensive disease spectrum to validate its performance in staging, classification, and differential diagnosis.

## Conclusion

In summary, our findings demonstrate a significant reduction in gut microbiota diversity as LC progresses. We identified 8 genera—*Veillonella*, *Lachnospira*, *Romboutsia*, *Akkermansia*, *Erysipelatoclostridium*, *Prevotella*, *UCG.005*, and *Streptococcus*—as key features for predicting LC and used them to train ML models. Among these models, the RF model showed superior discrimination, calibration, and clinical applicability compared to other ML models, effectively distinguishing LC individuals from healthy ones. Combined with SHAP analysis and deployment as a web application, our model has the potential to provide decision support for healthcare professionals and shows promise as a valuable auxiliary tool for LC screening and early clinical intervention.

## Supplementary Information

Supplementary Material 1. **Figure S1** The forest plot shows the outcomes of a meta-analysis performed on the alpha-diversity of each parameter in both the LC and HC groups. The pooled results showed a significant decrease in alpha diversity in LC compared to the HC group. *LC* liver cirrhosis, *HC* healthy control. **Figure S2** The forest plot shows the outcomes of a meta-analysis performed on the alpha-diversity of each parameter in both the LC and Hepatocellular carcinoma (HCC) groups. The pooled results showed no significant decrease in alpha diversity in HCC compared to the LC group. *LC* liver cirrhosis, *HCC* Hepatocellular carcinoma. **Figure S3** The gut microbial beta diversity changed as LC progressed based on NMDS analysis. The NMDS analysis showed that the gut microbiome composition was significantly different based on the ANOSIM test in the datasets of PRJNA1208993 (Gulyaeva et al., 2025), PRJNA558158 (Chen et al., 2020), PRJEB28350 (Caussy et al., 2019), PRJNA838083 (Li et al., 2022), PRJNA540574 (Zheng et al., 2020), PRJNA471972 (Iebba et al., 2018), PRJEB32568 (NA), and PRJNA784025 (Sun et al., 2025). * *P*<0.05, ** *P*<0.01, *** *P*<0.001. *NS* Not Statistically Significant, *LC* liver cirrhosis, *NA* Not Applicable. **Figure S4** The gut microbial beta diversity changed as LC progressed based on PCoA analysis. The PCoA analysis showed that the gut microbiome composition was significantly different based on the PERMANOVA test in the datasets of PRJNA1208993 (Gulyaeva et al., 2025), PRJNA558158 (Chen et al., 2020), PRJNA838083 (Li et al., 2022), PRJNA471972 (Iebba et al., 2018), PRJEB32568 (NA), PRJNA784025 (Sun et al., 2025), and PRJNA1259947 (Shi et al., 2025). * *P*<0.05, ** *P*<0.01, *** *P*<0.001. *NS* Not Statistically Significant, *LC* liver cirrhosis, *HCC* Hepatocellular carcinoma, *NA* Not Applicable. **Figure S5** The Venn diagram shows the intersection of the ASV for each group in each study. Based on Venn diagrams showing ASV intersections between groups, there were differences between groups based on ASV levels, including total ASV abundance, shared ASV and unique ASV in the liver cirrhosis group. **Figure S6** Analysis of KEGG pathways with significantly different relative abundances between HC and LC groups. *HC* healthy control, *LC* liver cirrhosis. **Figure S7** Analysis of KEGG pathways with significantly different relative abundances between LC and HCC groups. *LC* liver cirrhosis, *HCC* Hepatocellular carcinoma. **Figure S8** Comparison of PLSDAbatch before and after batch correction. [A] PCA scatter plot. Before correction (left): samples from different batches are clearly separated in PC space, indicating a strong batch effect. After correction (right): samples from different batches show high overlap, indicating effective batch effect removal. [B] Quantification of batch effect reduction by average Euclidean distance. Inter-batch distance decreased from 14.676 to 0.000, confirming complete removal of batch effects. Inter-condition distance changed slightly from 3.405 to 2.700, showing that biological differences between conditions are preserved. **Figure S9** Correlation Heatmap: As indicated by the light-colored cells, with blue and red areas representing negative and positive correlations, respectively. **Figure S10** The integration of LEfSe-based methods (LDA score ≥ 2 and p < 0.05) identifies crucial genera that significantly increase in the occurrence of HCC. *HCC* Hepatocellular carcinoma, * *P*<0.05, ** *P*<0.01, *** *P*<0.001. **Figure S11** Evaluation of 10 machine learning models using five‑fold cross‑validation. [A] Receiver operating characteristic curves; [B] Calibration curves; [C] Decision curve analysis. In the five‑fold cross‑validation, the integrated dataset was randomly partitioned into five equal‑sized folds. Each fold was held out once as the validation set while the remaining four folds were used for training, and this process was repeated five times. **Figure S12** Receiver operating characteristic curves of 10 machine learning models in the LODO analysis. In the LODO analysis, all datasets except one were used for training, and the left-out dataset was then used for testing. *LODO* leave-one-dataset-out. **Figure S13** Calibration curves of 10 machine learning models in the LODO analysis. In the LODO analysis, all datasets except one were used for training, and the left-out dataset was then used for testing. *LODO* leave-one-dataset-out. **Figure S14** Decision curve analysis of 10 machine learning models in the LODO analysis. In the LODO analysis, all datasets except one were used for training, and the left-out dataset was then used for testing. *LODO* leave-one-dataset-out. **Table S1**. Search Strategy. **Table S2**. The reads were trimmed using the following parameters in the DADA2 plugin for each study. **Table S3**. The R code for arcsine square root transformation and z-score normalization. **Table S4**. Hyperparameter grid of 10 ML models. **Table S5**. Diagnostic methods and criteria for LC in the included studies

## Data Availability

All data generated or analysed during this study are included in this published article [and its supplementary information files].

## Abbreviations

16S rRNA: 16S ribosomal RNA
AdaBoost: Adaptive Boosting
AI: Artificial intelligence
ANOSIM: Analysis of similarities
ASV: Amplicon sequence variant
AUC: Area under the receiver operating characteristic curve
CI: Confidence intervals
DADA2: Divisive Amplicon Denoising Algorithm 2
DCA: Decision curve analysis
DT: Decision Tree
GNB: Gaussian Naive Bayes
HC: Healthy control
HCC: Hepatocellular carcinoma
IQR: Interquartile range
KEGG: Kyoto Encyclopedia of Genes and Genomes
KNN: K-Nearest Neighbors
LASSO: Least Absolute Shrinkage and Selection Operator
LC: liver cirrhosis
LEfSe: linear discriminant analysis effect size
LGBM: Light Gradient Boosting Machine
LODO: Leave-one-dataset-out
LR: Logistic Regression
MCC: Matthews Correlation Coefficient
ML: Machine learning
MLP: Multilayer Perceptron
NCBI: National Center for Biotechnology Information
NMDS: Non-metric multidimensional scaling
PCoA: Principal coordinate analysis
PD: Phylogenetic diversity
PERMANOVA: Permutational multivariate analysis of variance
QIIME 2: Quantitative Insights Into Microbial Ecology version 2
RF: Random Forest
SD: Standard deviation
SHAP: SHapley Additive exPlanations
SMD: Standardized mean differences
SRA: Sequence Read Archive
SVM: Support Vector Machine
XGB: Extreme Gradient Boosting
