# Supplementary material for: Machine learning-driven clinical decision support for liver cirrhosis: a gut microbiome-based web prediction model with explainable AI integration
Source: BMC Gastroenterol. 2026 May 6;26:318. doi: 10.1186/s12876-026-04890-7 (PMC13192034; doi:10.1186/s12876-026-04890-7)
Supplement: Supplementary file 1 — Supplementary Material 1. Figure S1 The forest plot shows the outcomes of a meta-analysis performed on the alpha-diversity of each parameter in both the LC and HC groups. The pooled results showed a significant decrease in alpha diversity in LC compared to the HC group. LC liver cirrhosis, HC healthy control. Figure S2 The forest plot shows the outcomes of a meta-analysis performed on the alpha-diversity of each parameter in both the LC and Hepatocellular carcinoma (HCC) groups. The pooled results showed no significant decrease in alpha diversity in HCC compared to the LC group. LC liver cirrhosis, HCC Hepatocellular carcinoma. Figure S3 The gut microbial beta diversity changed as LC progressed based on NMDS analysis. The NMDS analysis showed that the gut microbiome composition was significantly different based on the ANOSIM test in the datasets of PRJNA1208993 (Gulyaeva et al., 2025), PRJNA558158 (Chen et al., 2020), PRJEB28350 (Caussy et al., 2019), PRJNA838083 (Li et al., 2022), PRJNA540574 (Zheng et al., 2020), PRJNA471972 (Iebba et al., 2018), PRJEB32568 (NA), and PRJNA784025 (Sun et al., 2025). * P<0.05, ** P<0.01, *** P<0.001. NS Not Statistically Significant, LC liver cirrhosis, NA Not Applicable. Figure S4 The gut microbial beta diversity changed as LC progressed based on PCoA analysis. The PCoA analysis showed that the gut microbiome composition was significantly different based on the PERMANOVA test in the datasets of PRJNA1208993 (Gulyaeva et al., 2025), PRJNA558158 (Chen et al., 2020), PRJNA838083 (Li et al., 2022), PRJNA471972 (Iebba et al., 2018), PRJEB32568 (NA), PRJNA784025 (Sun et al., 2025), and PRJNA1259947 (Shi et al., 2025). * P<0.05, ** P<0.01, *** P<0.001. NS Not Statistically Significant, LC liver cirrhosis, HCC Hepatocellular carcinoma, NA Not Applicable. Figure S5 The Venn diagram shows the intersection of the ASV for each group in each study. Based on Venn diagrams showing ASV intersections between groups, there were differences between g [file 12876_2026_4890_MOESM1_ESM.pdf]

Study or Subgroup	LC Mean	SD	Total	HC Mean	SD	Total	Weight	Std. Mean Difference IV, Random, 95% CI
**Observed**								
Gulyaeva et al., 2025	290.81	106.3436	21	263.06	58.5677	18	1.2%	0.31 [−0.32, 0.94]
Li et al., 2022	467.82	95.1054	17	594.71	119.9240	14	1.0%	−1.16 [−1.93, −0.38]
Zhong et al., 2021	89.10	33.3015	10	102.25	29.5294	20	1.0%	−0.42 [−1.18, 0.35]
Zheng et al., 2020	256.11	54.5984	19	340.92	54.1825	13	0.9%	−1.52 [−2.33, −0.71]
Chen et al., 2020	119.47	43.9760	17	143.55	61.6707	11	1.0%	−0.45 [−1.22, 0.32]
Caussy et al., 2019	122.83	65.7967	18	141.08	64.7169	90	1.4%	−0.28 [−0.79, 0.23]
Liu et al., 2018	214.04	22.5360	25	208.76	18.9523	17	1.2%	0.24 [−0.37, 0.86]
Iebba et al., 2018	63.39	62.6683	36	76.33	69.7801	18	1.3%	−0.20 [−0.76, 0.37]
NA, BioProject number: PRJEB32568	303.78	53.1784	9	221.75	19.6702	4	0.4%	1.64 [0.25, 3.04]
Sun et al., 2025	259.86	74.9995	73	324.54	64.5442	48	1.6%	−0.90 [−1.29, −0.52]
Shi et al., 2025	186.84	67.2813	64	214.14	62.3032	28	1.5%	−0.41 [−0.86, 0.04]
**Total (95% CI)**			**309**			**281**	**12.4%**	**−0.36 [−0.74, 0.02]**
Heterogeneity: Tau^2 = 0.290; Chi^2 = 35.56, df = 10 (P = 0.0001); I^2 = 72%								
Test for overall effect: Z = −1.87 (P = 0.0621)								
**Chao1**								
Gulyaeva et al., 2025	299.33	112.9627	21	266.54	59.5825	18	1.2%	0.35 [−0.29, 0.98]
Li et al., 2022	468.47	94.8247	17	594.91	120.0548	14	1.0%	−1.15 [−1.92, −0.38]
Zhong et al., 2021	89.10	33.3015	10	102.25	29.5294	20	1.0%	−0.42 [−1.18, 0.35]
Zheng et al., 2020	256.12	54.6300	19	340.92	54.1825	13	0.9%	−1.52 [−2.33, −0.71]
Chen et al., 2020	122.06	46.5611	17	150.34	66.8933	11	1.0%	−0.50 [−1.27, 0.27]
Caussy et al., 2019	124.31	66.8850	18	142.68	65.7376	90	1.4%	−0.28 [−0.78, 0.23]
Liu et al., 2018	214.04	22.5360	25	208.76	18.9523	17	1.2%	0.24 [−0.37, 0.86]
Iebba et al., 2018	63.39	62.6683	36	76.33	69.7801	18	1.3%	−0.20 [−0.76, 0.37]
NA, BioProject number: PRJEB32568	306.49	53.9165	9	223.58	20.7960	4	0.4%	1.63 [0.24, 3.02]
Sun et al., 2025	262.63	75.4984	73	329.32	65.2255	48	1.6%	−0.93 [−1.31, −0.54]
Shi et al., 2025	187.61	67.6251	64	215.33	62.6545	28	1.5%	−0.42 [−0.86, 0.03]
**Total (95% CI)**			**309**			**281**	**12.4%**	**−0.36 [−0.75, 0.02]**
Heterogeneity: Tau^2 = 0.302; Chi^2 = 36.54, df = 10 (P < 0.0001); I^2 = 73%								
Test for overall effect: Z = −1.84 (P = 0.0651)								
**ACE**								
Gulyaeva et al., 2025	296.50	110.8779	21	265.58	59.6233	18	1.2%	0.33 [−0.30, 0.97]
Li et al., 2022	467.96	95.1025	17	594.86	119.9539	14	1.0%	−1.16 [−1.93, −0.38]
Zhong et al., 2021	87.33	34.8210	10	105.88	28.5479	20	1.0%	−0.59 [−1.36, 0.19]
Zheng et al., 2020	256.13	54.6465	19	340.95	54.1302	13	0.9%	−1.52 [−2.33, −0.71]
Chen et al., 2020	122.23	45.9169	17	148.57	65.3952	11	1.0%	−0.47 [−1.24, 0.30]
Caussy et al., 2019	123.92	66.5124	18	142.50	65.4619	90	1.4%	−0.28 [−0.79, 0.23]
Liu et al., 2018	215.65	22.8817	25	211.56	18.2124	17	1.2%	0.19 [−0.43, 0.81]
Iebba et al., 2018	63.39	62.6683	36	76.37	69.8003	18	1.3%	−0.20 [−0.76, 0.37]
NA, BioProject number: PRJEB32568	305.77	53.7054	9	222.54	19.5597	4	0.4%	1.65 [0.25, 3.05]
Sun et al., 2025	261.97	75.2119	73	327.62	65.0508	48	1.6%	−0.91 [−1.30, −0.53]
Shi et al., 2025	187.42	67.4143	64	215.12	62.5832	28	1.5%	−0.42 [−0.86, 0.03]
**Total (95% CI)**			**309**			**281**	**12.4%**	**−0.38 [−0.76, −0.00]**
Heterogeneity: Tau^2 = 0.292; Chi^2 = 35.57, df = 10 (P < 0.0001); I^2 = 72%								
Test for overall effect: Z = −1.97 (P = 0.0492)								
**Shannon**								
Gulyaeva et al., 2025	5.00	0.5125	21	5.07	0.2740	18	1.2%	−0.17 [−0.80, 0.46]
Li et al., 2022	5.60	0.3549	17	5.98	0.3321	14	1.0%	−1.07 [−1.83, −0.31]
Zhong et al., 2021	3.90	0.6374	10	4.03	0.5800	20	1.0%	−0.21 [−0.97, 0.55]
Zheng et al., 2020	4.95	0.5268	19	5.46	0.3209	13	1.0%	−1.08 [−1.84, −0.32]
Chen et al., 2020	4.25	0.4639	17	4.33	0.5618	11	1.0%	−0.16 [−0.92, 0.60]
Caussy et al., 2019	2.81	0.7532	18	2.96	1.1119	90	1.4%	−0.14 [−0.65, 0.37]
Liu et al., 2018	5.00	0.1596	25	5.00	0.1081	17	1.2%	−0.02 [−0.64, 0.60]
Iebba et al., 2018	3.12	1.1416	36	3.24	1.0723	18	1.3%	−0.11 [−0.67, 0.46]
NA, BioProject number: PRJEB32568	4.06	0.5434	9	4.00	0.1878	4	0.6%	0.11 [−1.07, 1.29]
Sun et al., 2025	3.77	0.7363	73	4.07	0.4787	48	1.7%	−0.47 [−0.83, −0.10]
Shi et al., 2025	3.64	0.7345	64	3.84	0.5487	28	1.5%	−0.28 [−0.73, 0.17]
**Total (95% CI)**			**309**			**281**	**12.6%**	**−0.33 [−0.50, −0.15]**
Heterogeneity: Tau^2 = < 0.001; Chi^2 = 11.14, df = 10 (P = 0.3468); I^2 = 10%								
Test for overall effect: Z = −3.61 (P = 0.0003)								
**Simpson**								
Gulyaeva et al., 2025	0.99	0.0100	21	0.99	0.0036	18	1.2%	−0.41 [−1.05, 0.23]
Li et al., 2022	0.99	0.0028	17	1.00	0.0020	14	1.0%	−0.93 [−1.68, −0.18]
Zhong et al., 2021	0.96	0.0273	10	0.97	0.0261	20	1.0%	−0.09 [−0.85, 0.67]
Zheng et al., 2020	0.98	0.0124	19	0.99	0.0049	13	1.0%	−0.82 [−1.56, −0.09]
Chen et al., 2020	0.98	0.0164	17	0.98	0.0180	11	1.0%	0.02 [−0.74, 0.78]
Caussy et al., 2019	0.84	0.0990	18	0.81	0.2300	90	1.4%	0.12 [−0.39, 0.62]
Liu et al., 2018	0.99	0.0032	25	0.99	0.0018	17	1.2%	−0.10 [−0.72, 0.52]
Iebba et al., 2018	0.89	0.1077	36	0.90	0.1030	18	1.3%	−0.05 [−0.61, 0.52]
NA, BioProject number: PRJEB32568	0.95	0.0342	9	0.96	0.0138	4	0.6%	−0.30 [−1.48, 0.89]
Sun et al., 2025	0.92	0.0859	73	0.94	0.0424	48	1.7%	−0.31 [−0.68, 0.06]
Shi et al., 2025	0.93	0.0574	64	0.94	0.0352	28	1.5%	−0.24 [−0.68, 0.21]
**Total (95% CI)**			**309**			**281**	**12.7%**	**−0.25 [−0.42, −0.07]**
Heterogeneity: Tau^2 = 0; Chi^2 = 9.26, df = 10 (P = 0.5076); I^2 = 0%								
Test for overall effect: Z = −2.75 (P = 0.0060)								
**InvSimpson**								
Gulyaeva et al., 2025	104.36	44.8201	21	116.35	36.4747	18	1.2%	−0.29 [−0.92, 0.35]
Li et al., 2022	188.73	92.4393	17	291.13	116.6827	14	1.0%	−0.96 [−1.71, −0.21]
Zhong et al., 2021	42.12	25.1787	10	43.01	21.5149	20	1.0%	−0.04 [−0.80, 0.72]
Zheng et al., 2020	103.18	62.0458	19	175.36	62.7266	13	1.0%	−1.13 [−1.89, −0.36]
Chen et al., 2020	55.93	24.7843	17	62.17	34.5156	11	1.0%	−0.21 [−0.97, 0.55]
Caussy et al., 2019	10.16	8.7224	18	14.91	12.0216	90	1.4%	−0.41 [−0.92, 0.10]
Liu et al., 2018	110.09	24.3183	25	109.39	19.6982	17	1.2%	0.03 [−0.59, 0.65]
Iebba et al., 2018	25.90	25.2947	36	27.32	28.5808	18	1.3%	−0.05 [−0.62, 0.51]
NA, BioProject number: PRJEB32568	29.24	16.8738	9	27.43	8.2530	4	0.6%	0.11 [−1.07, 1.29]
Sun et al., 2025	23.42	15.9350	73	26.03	14.8477	48	1.7%	−0.17 [−0.53, 0.20]
Shi et al., 2025	23.18	13.9171	64	24.29	12.7610	28	1.5%	−0.08 [−0.53, 0.36]
**Total (95% CI)**			**309**			**281**	**12.7%**	**−0.25 [−0.42, −0.07]**
Heterogeneity: Tau^2 = < 0.001; Chi^2 = 11.56, df = 10 (P = 0.3154); I^2 = 14%								
Test for overall effect: Z = −2.76 (P = 0.0058)								
**Fisher**								
Gulyaeva et al., 2025	65.64	29.5912	21	56.70	16.0546	18	1.2%	0.36 [−0.28, 1.00]
Li et al., 2022	81.52	19.8112	17	109.17	26.3429	14	1.0%	−1.17 [−1.94, −0.40]
Zhong et al., 2021	14.54	6.3680	10	17.04	5.7370	20	1.0%	−0.41 [−1.18, 0.36]
Zheng et al., 2020	41.94	10.6304	19	58.99	11.1062	13	0.9%	−1.54 [−2.35, −0.72]
Chen et al., 2020	28.29	13.9919	17	36.42	21.0084	11	1.0%	−0.46 [−1.23, 0.31]
Caussy et al., 2019	17.43	10.9231	18	20.38	10.8589	90	1.4%	−0.27 [−0.78, 0.24]
Liu et al., 2018	34.99	4.3651	25	33.95	3.6810	17	1.2%	0.25 [−0.37, 0.87]
Iebba et al., 2018	15.56	15.2186	36	18.60	17.1882	18	1.3%	−0.19 [−0.76, 0.38]
NA, BioProject number: PRJEB32568	43.56	8.8273	9	30.14	3.0868	4	0.4%	1.62 [0.23, 3.01]
Sun et al., 2025	36.66	12.1580	73	47.28	10.9750	48	1.6%	−0.90 [−1.28, −0.52]
Shi et al., 2025	25.93	10.7232	64	30.24	10.0565	28	1.5%	−0.41 [−0.85, 0.04]
**Total (95% CI)**			**309**			**281**	**12.4%**	**−0.35 [−0.74, 0.03]**
Heterogeneity: Tau^2 = 0.307; Chi^2 = 36.57, df = 10 (P < 0.0001); I^2 = 73%								
Test for overall effect: Z = −1.80 (P = 0.0723)								
**PD**								
Gulyaeva et al., 2025	17.75	7.3966	21	18.59	8.0122	18	1.2%	−0.11 [−0.74, 0.52]
Li et al., 2022	23.42	3.2767	17	25.35	3.7134	14	1.0%	−0.54 [−1.26, 0.18]
Zhong et al., 2021	8.97	2.7490	10	9.93	2.1378	20	1.0%	−0.40 [−1.16, 0.37]
Zheng et al., 2020	9.78	1.1054	19	11.18	0.8386	13	0.9%	−1.36 [−2.15, −0.57]
Chen et al., 2020	7.62	6.1315	17	6.02	3.2435	11	1.0%	0.30 [−0.47, 1.06]
Caussy et al., 2019	12.38	3.7818	18	13.68	4.1469	90	1.4%	−0.32 [−0.82, 0.19]
Liu et al., 2018	11.84	1.4184	25	11.21	1.0300	17	1.2%	0.49 [−0.14, 1.11]
Iebba et al., 2018	34.16	45.9197	36	46.17	37.2141	18	1.3%	−0.27 [−0.84, 0.29]
NA, BioProject number: PRJEB32568	16.10	1.6691	9	12.77	1.5237	4	0.4%	1.90 [0.44, 3.36]
Sun et al., 2025	16.24	4.6765	73	16.00	3.5567	48	1.7%	0.06 [−0.31, 0.42]
Shi et al., 2025	17.34	3.9074	64	16.66	2.6160	28	1.5%	0.19 [−0.26, 0.63]
**Total (95% CI)**			**309**			**281**	**12.5%**	**−0.09 [−0.40, 0.22]**
Heterogeneity: Tau^2 = 0.165; Chi^2 = 26.65, df = 10 (P = 0.0030); I^2 = 62%								
Test for overall effect: Z = −0.56 (P = 0.5766)								
**Total (95% CI)**			**2472**			**2248**	**100.0%**	**−0.31 [−0.41, −0.21]**
Heterogeneity: Tau^2 = 0.130; Chi^2 = 218.28, df = 87 (P < 0.0001); I^2 = 60%								
Test for overall effect: Z = −5.95 (P < 0.0001)								
Test for subgroup differences: Chi^2 = 2.73, df = 7 (P = 0.9089)								

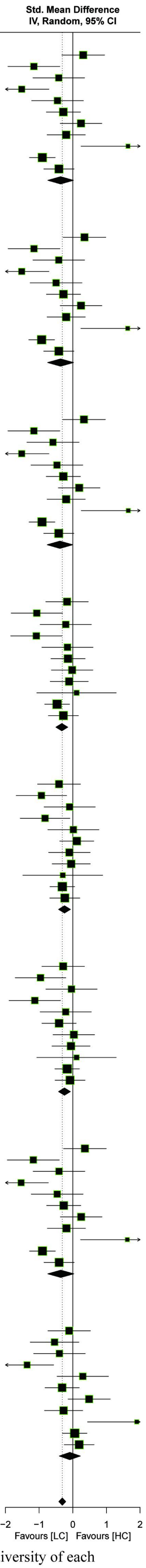

Figure S1 The forest plot shows the outcomes of a meta-analysis performed on the alpha-diversity of each parameter in both the LC and HC groups. The pooled results showed a significant decrease in alpha diversity in LC compared to the HC group. *LC* liver cirrhosis, *HC* healthy control.

Study or Subgroup	HCC Mean	SD	Total	LC Mean	SD	Total	Weight	Std. Mean Difference IV, Random, 95% CI
**Observed**								
Li et al., 2022	467.82	95.1054	17	444.85	122.5240	13	4.3%	0.21 [−0.52, 0.93]
Zheng et al., 2020	256.11	54.5984	19	331.92	68.0214	65	4.9%	−1.15 [−1.69, −0.61]
NA, BioProject number: PRJEB32568	303.78	53.1784	9	235.50	92.9059	6	3.2%	0.90 [−0.20, 2.00]
**Total (95% CI)**			**45**			**84**	**12.4%**	**−0.08 [−1.27, 1.11]**
Heterogeneity: $Tau^2 = 0.937$; $Chi^2 = 15.43$, df = 2 (P = 0.0004); $I^2 = 87\%$								
Test for overall effect: Z = −0.13 (P = 0.8939)								
**Chao1**								
Li et al., 2022	468.47	94.8247	17	448.15	128.4883	13	4.3%	0.18 [−0.54, 0.90]
Zheng et al., 2020	256.12	54.6300	19	332.01	68.0964	65	4.9%	−1.15 [−1.69, −0.61]
NA, BioProject number: PRJEB32568	306.49	53.9165	9	237.20	93.3165	6	3.2%	0.91 [−0.19, 2.01]
**Total (95% CI)**			**45**			**84**	**12.4%**	**−0.09 [−1.28, 1.10]**
Heterogeneity: $Tau^2 = 0.931$; $Chi^2 = 15.2$, df = 2 (P = 0.0005); $I^2 = 87\%$								
Test for overall effect: Z = −0.15 (P = 0.8828)								
**ACE**								
Li et al., 2022	467.96	95.1025	17	447.24	126.7331	13	4.3%	0.18 [−0.54, 0.91]
Zheng et al., 2020	256.13	54.6465	19	331.99	67.9894	65	4.9%	−1.15 [−1.69, −0.61]
NA, BioProject number: PRJEB32568	305.77	53.7054	9	236.73	93.2323	6	3.2%	0.91 [−0.19, 2.01]
**Total (95% CI)**			**45**			**84**	**12.4%**	**−0.09 [−1.28, 1.10]**
Heterogeneity: $Tau^2 = 0.933$; $Chi^2 = 15.25$, df = 2 (P = 0.0005); $I^2 = 87\%$								
Test for overall effect: Z = −0.15 (P = 0.8841)								
**Shannon**								
Li et al., 2022	5.60	0.3549	17	5.51	0.4865	13	4.3%	0.22 [−0.50, 0.95]
Zheng et al., 2020	4.95	0.5268	19	5.32	0.4267	65	4.9%	−0.82 [−1.34, −0.29]
NA, BioProject number: PRJEB32568	4.06	0.5434	9	3.66	0.8207	6	3.3%	0.56 [−0.50, 1.61]
**Total (95% CI)**			**45**			**84**	**12.6%**	**−0.09 [−0.93, 0.75]**
Heterogeneity: $Tau^2 = 0.399$; $Chi^2 = 8.19$, df = 2 (P = 0.0166); $I^2 = 76\%$								
Test for overall effect: Z = −0.22 (P = 0.8266)								
**Simpson**								
Li et al., 2022	0.99	0.0028	17	0.99	0.0049	13	4.3%	0.20 [−0.52, 0.93]
Zheng et al., 2020	0.98	0.0124	19	0.99	0.0091	65	4.9%	−0.57 [−1.08, −0.05]
NA, BioProject number: PRJEB32568	0.95	0.0342	9	0.93	0.0543	6	3.3%	0.53 [−0.52, 1.59]
**Total (95% CI)**			**45**			**84**	**12.6%**	**−0.05 [−0.71, 0.62]**
Heterogeneity: $Tau^2 = 0.200$; $Chi^2 = 4.96$, df = 2 (P = 0.0838); $I^2 = 60\%$								
Test for overall effect: Z = −0.14 (P = 0.8913)								
**InvSimpson**								
Li et al., 2022	188.73	92.4393	17	182.27	82.5557	13	4.3%	0.07 [−0.65, 0.79]
Zheng et al., 2020	103.18	62.0458	19	145.56	71.2259	65	4.9%	−0.61 [−1.13, −0.09]
NA, BioProject number: PRJEB32568	29.24	16.8738	9	22.42	15.2379	6	3.4%	0.39 [−0.65, 1.44]
**Total (95% CI)**			**45**			**84**	**12.6%**	**−0.16 [−0.76, 0.44]**
Heterogeneity: $Tau^2 = 0.141$; $Chi^2 = 4.01$, df = 2 (P = 0.1344); $I^2 = 50\%$								
Test for overall effect: Z = −0.51 (P = 0.6072)								
**Fisher**								
Li et al., 2022	81.52	19.8112	17	77.06	25.2532	13	4.3%	0.19 [−0.53, 0.92]
Zheng et al., 2020	41.94	10.6304	19	57.27	14.0222	65	4.9%	−1.14 [−1.68, −0.60]
NA, BioProject number: PRJEB32568	43.56	8.8273	9	32.77	14.4373	6	3.2%	0.90 [−0.20, 1.99]
**Total (95% CI)**			**45**			**84**	**12.4%**	**−0.08 [−1.26, 1.09]**
Heterogeneity: $Tau^2 = 0.912$; $Chi^2 = 15.04$, df = 2 (P = 0.0005); $I^2 = 87\%$								
Test for overall effect: Z = −0.14 (P = 0.8884)								
**PD**								
Li et al., 2022	23.42	3.2767	17	21.97	6.6163	13	4.3%	0.28 [−0.44, 1.01]
Zheng et al., 2020	9.78	1.1054	19	11.63	2.8223	65	4.9%	−0.72 [−1.24, −0.20]
NA, BioProject number: PRJEB32568	16.10	1.6691	9	13.30	3.8967	6	3.2%	0.96 [−0.15, 2.06]
**Total (95% CI)**			**45**			**84**	**12.5%**	**0.08 [−0.87, 1.03]**
Heterogeneity: $Tau^2 = 0.545$; $Chi^2 = 9.68$, df = 2 (P = 0.0079); $I^2 = 79\%$								
Test for overall effect: Z = 0.17 (P = 0.8628)								
**Total (95% CI)**			**360**			**672**	**100.0%**	**−0.09 [−0.39, 0.20]**
Heterogeneity: $Tau^2 = 0.389$; $Chi^2 = 90.17$, df = 23 (P < 0.0001); $I^2 = 74\%$								
Test for overall effect: Z = −0.62 (P = 0.5341)								
Test for subgroup differences: $Chi^2 = 0.19$, df = 7 (P = 1.0000)								

Std. Mean Difference IV, Random, 95% CI

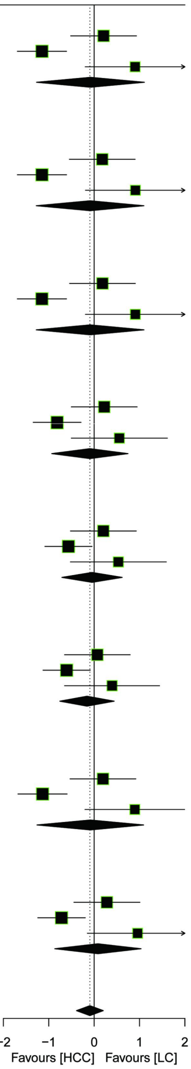

Figure S2 The forest plot shows the outcomes of a meta-analysis performed on the alpha-diversity of each parameter in both the LC and Hepatocellular carcinoma (HCC) groups. The pooled results showed no significant decrease in alpha diversity in HCC compared to the LC group. *LC* liver cirrhosis, *HCC* Hepatocellular carcinoma.

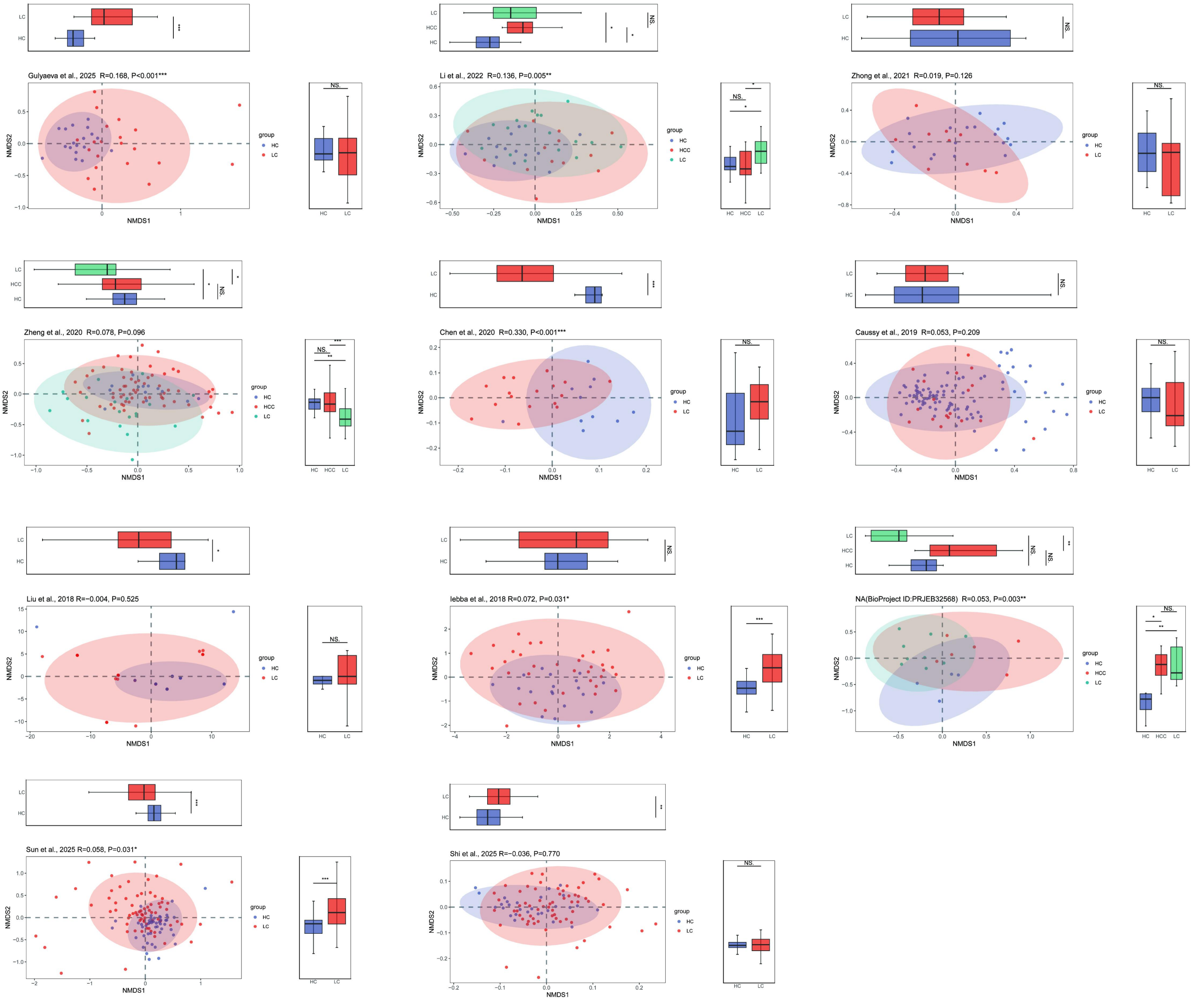

Figure S3 The gut microbial beta diversity changed as LC progressed based on NMDS analysis. The NMDS analysis showed that the gut microbiome composition was significantly different based on the ANOSIM test in the datasets of PRJNA1208993 (Gulyaeva et al., 2025), PRJNA558158 (Chen et al., 2020), PRJEB28350 (Caussy et al., 2019), PRJNA838083 (Li et al., 2022), PRJNA540574 (Zheng et al., 2020), PRJNA471972 (Iebba et al., 2018), PRJEB32568 (NA), and PRJNA784025 (Sun et al., 2025). * *P*<0.05, ** *P*<0.01, *** *P*<0.001. *NS* Not Statistically Significant, *LC* liver cirrhosis, *NA* Not Applicable.

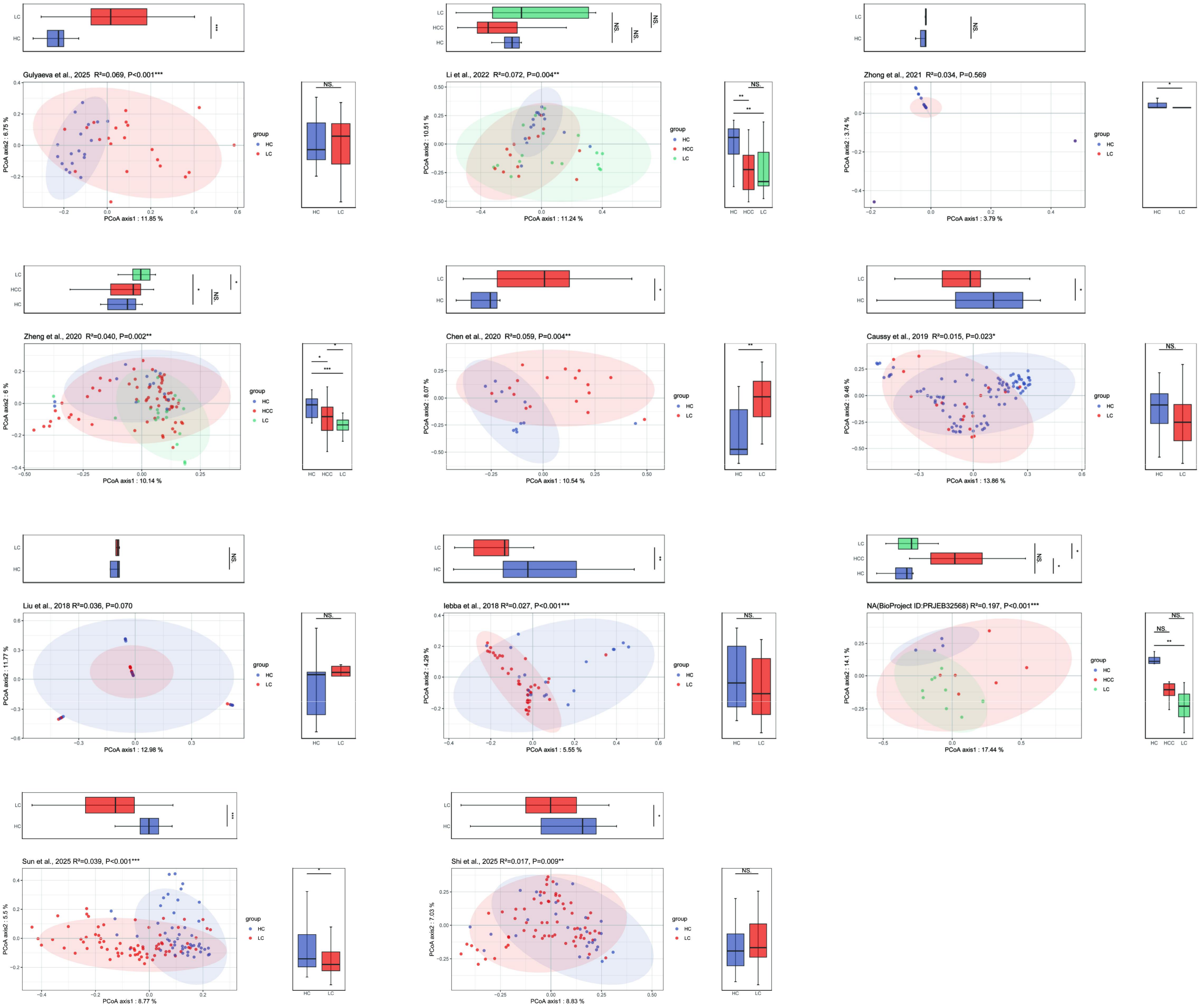

Figure S4 The gut microbial beta diversity changed as LC progressed based on PCoA analysis. The PCoA analysis showed that the gut microbiome composition was significantly different based on the PERMANOVA test in the datasets of PRJNA1208993 (Gulyaeva et al., 2025), PRJNA558158 (Chen et al., 2020), PRJNA838083 (Li et al., 2022), PRJNA471972 (Iebba et al., 2018), PRJEB32568 (NA), PRJNA784025 (Sun et al., 2025), and PRJNA1259947 (Shi et al., 2025). * *P*<0.05, ** *P*<0.01, *** *P*<0.001. *NS* Not Statistically Significant, *LC* liver cirrhosis, *HCC* Hepatocellular carcinoma, *NA* Not Applicable.

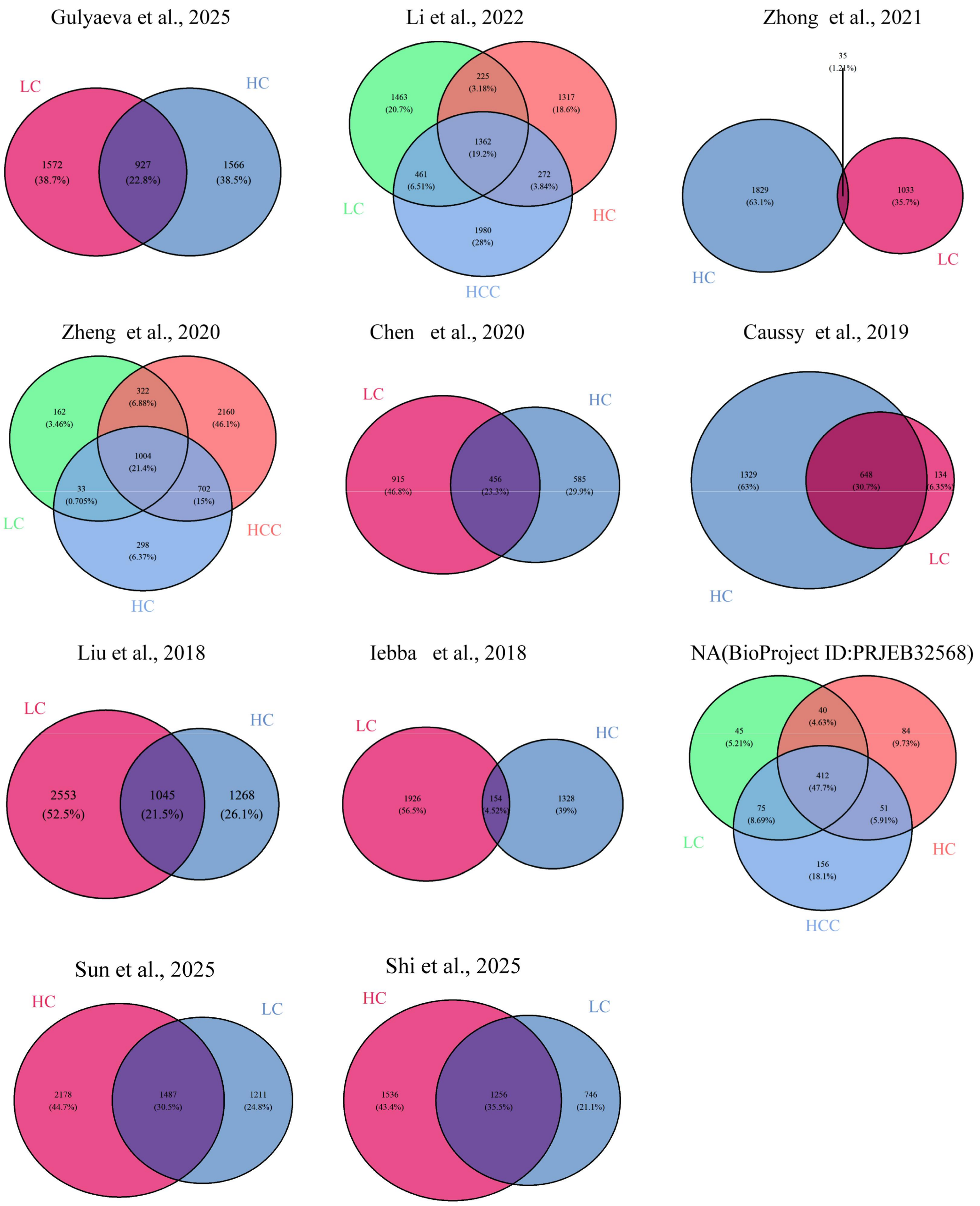

Figure S5 The Venn diagram shows the intersection of the ASV for each group in each study. Based on Venn diagrams showing ASV intersections between groups, there were differences between groups based on ASV levels, including total ASV abundance, shared ASV and unique ASV in the liver cirrhosis group.

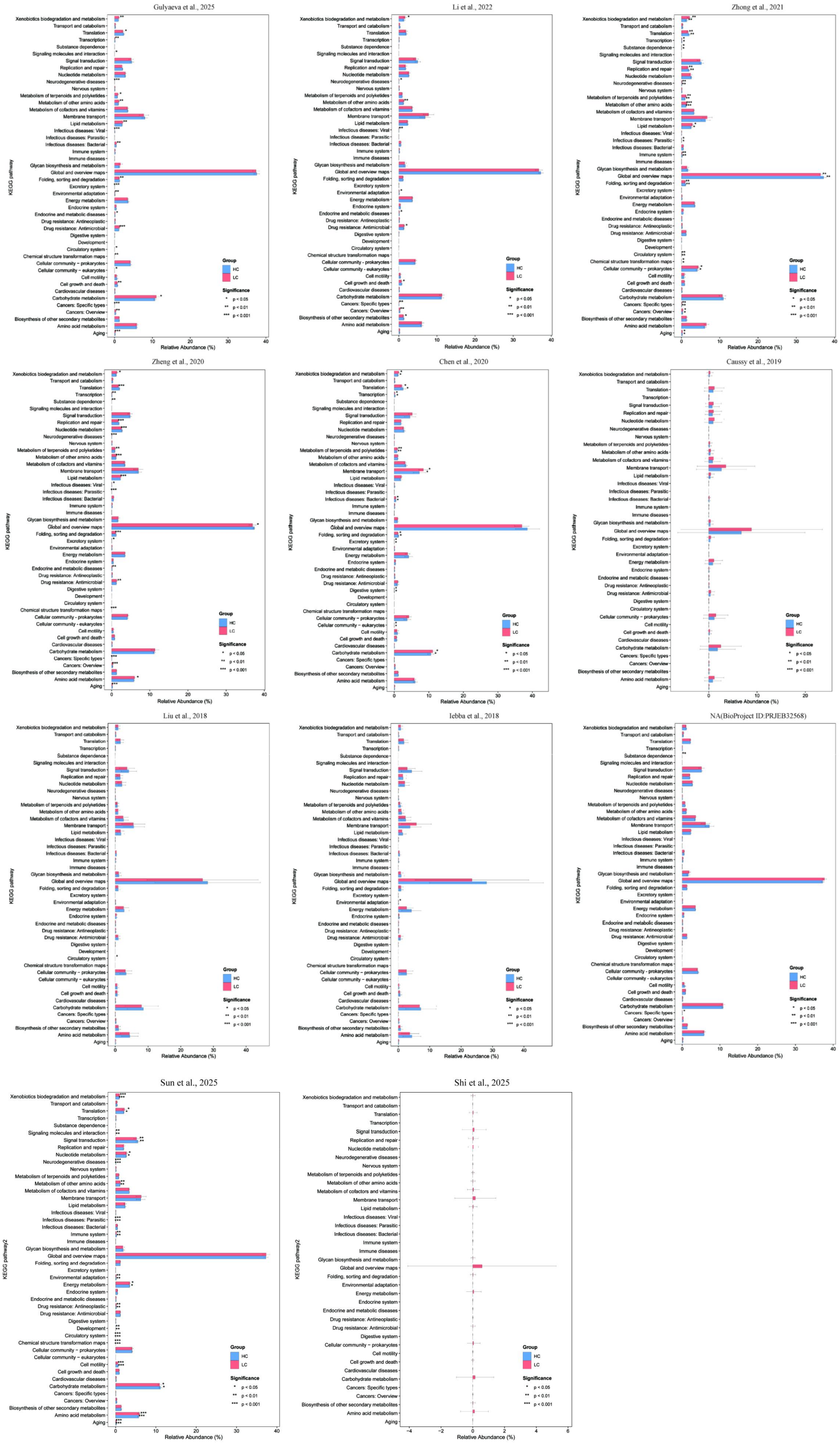

Figure S6 Analysis of KEGG pathways with significantly different relative abundances between HC and LC groups. *HC* healthy control, *LC* liver cirrhosis.

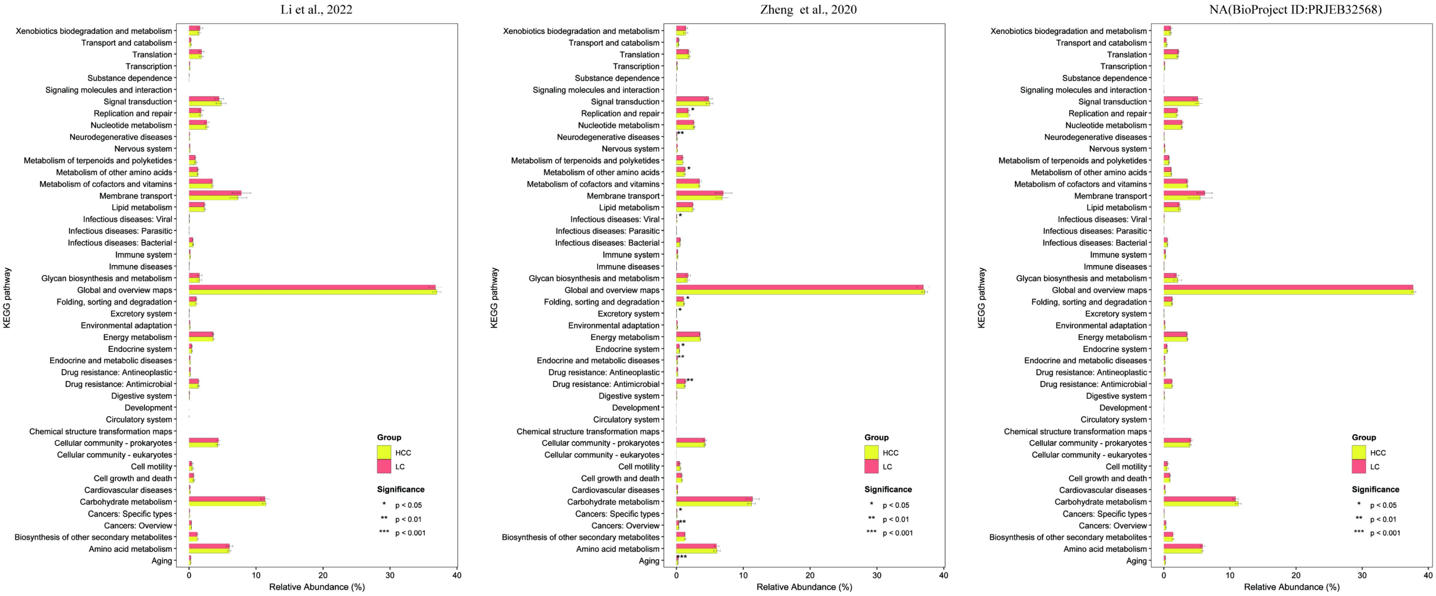

Figure S7 Analysis of KEGG pathways with significantly different relative abundances between LC and HCC groups. *LC* liver cirrhosis, *HCC* Hepatocellular carcinoma.

A

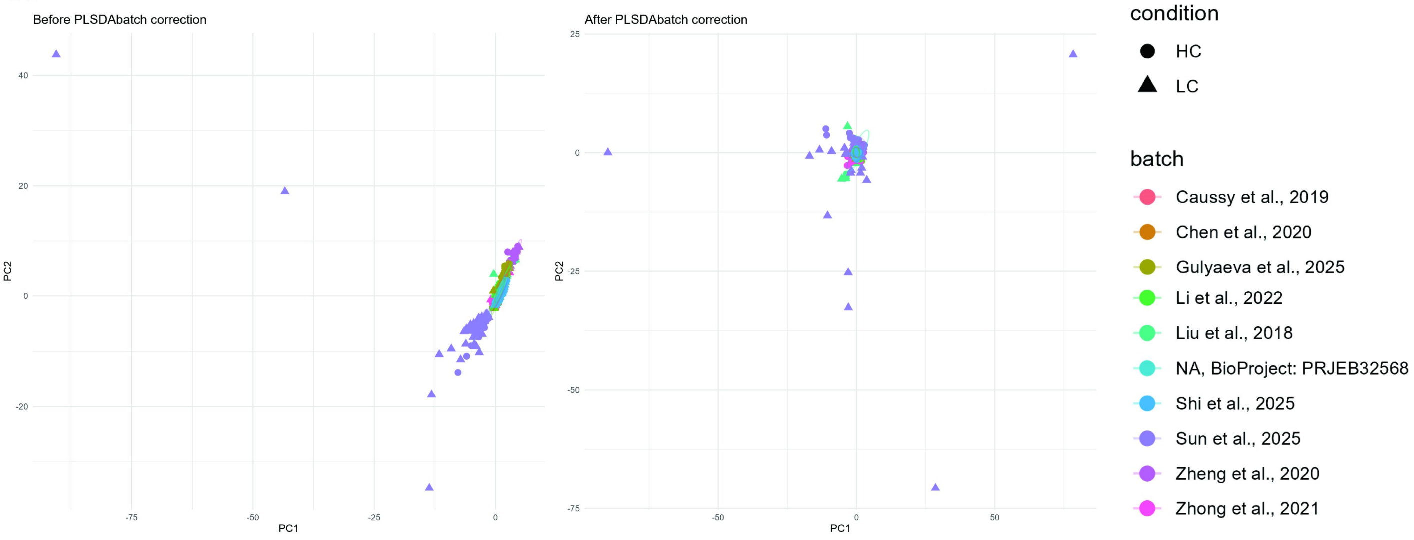

B

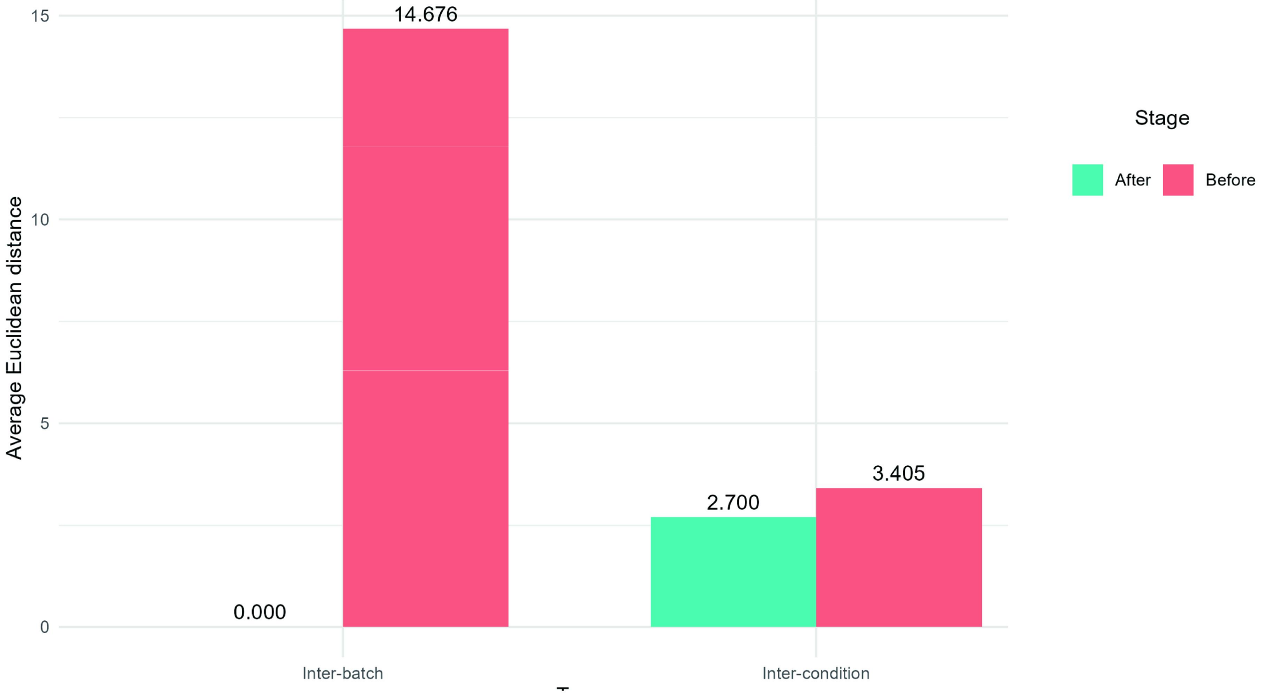

Figure S8 Comparison of PLSDAbatch before and after batch correction. [A] PCA scatter plot. Before correction (left): samples from different batches are clearly separated in PC space, indicating a strong batch effect. After correction (right): samples from different batches show high overlap, indicating effective batch effect removal. [B] Quantification of batch effect reduction by average Euclidean distance. Inter-batch distance decreased from 14.676 to 0.000, confirming complete removal of batch effects. Inter-condition distance changed slightly from 3.405 to 2.700, showing that biological differences between conditions are preserved.

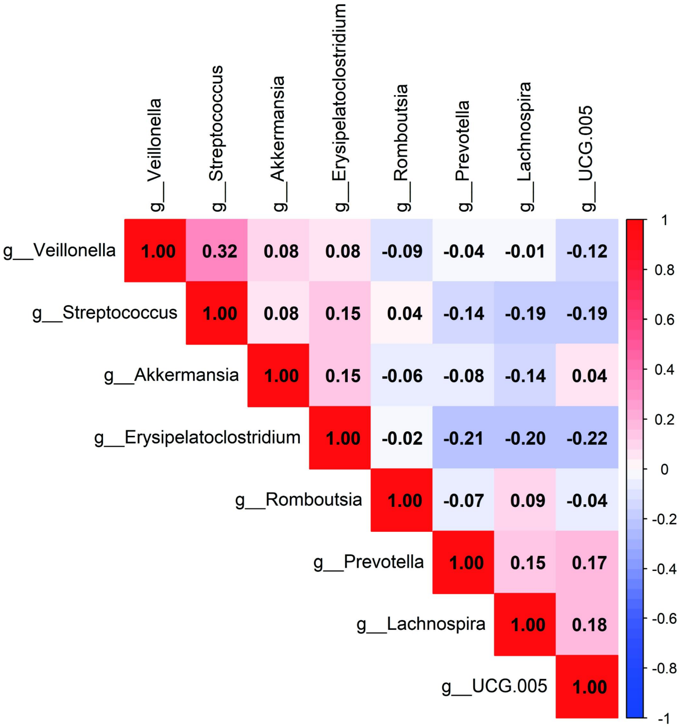

Figure S9 Correlation Heatmap: As indicated by the light-colored cells, with blue and red areas representing negative and positive correlations, respectively.

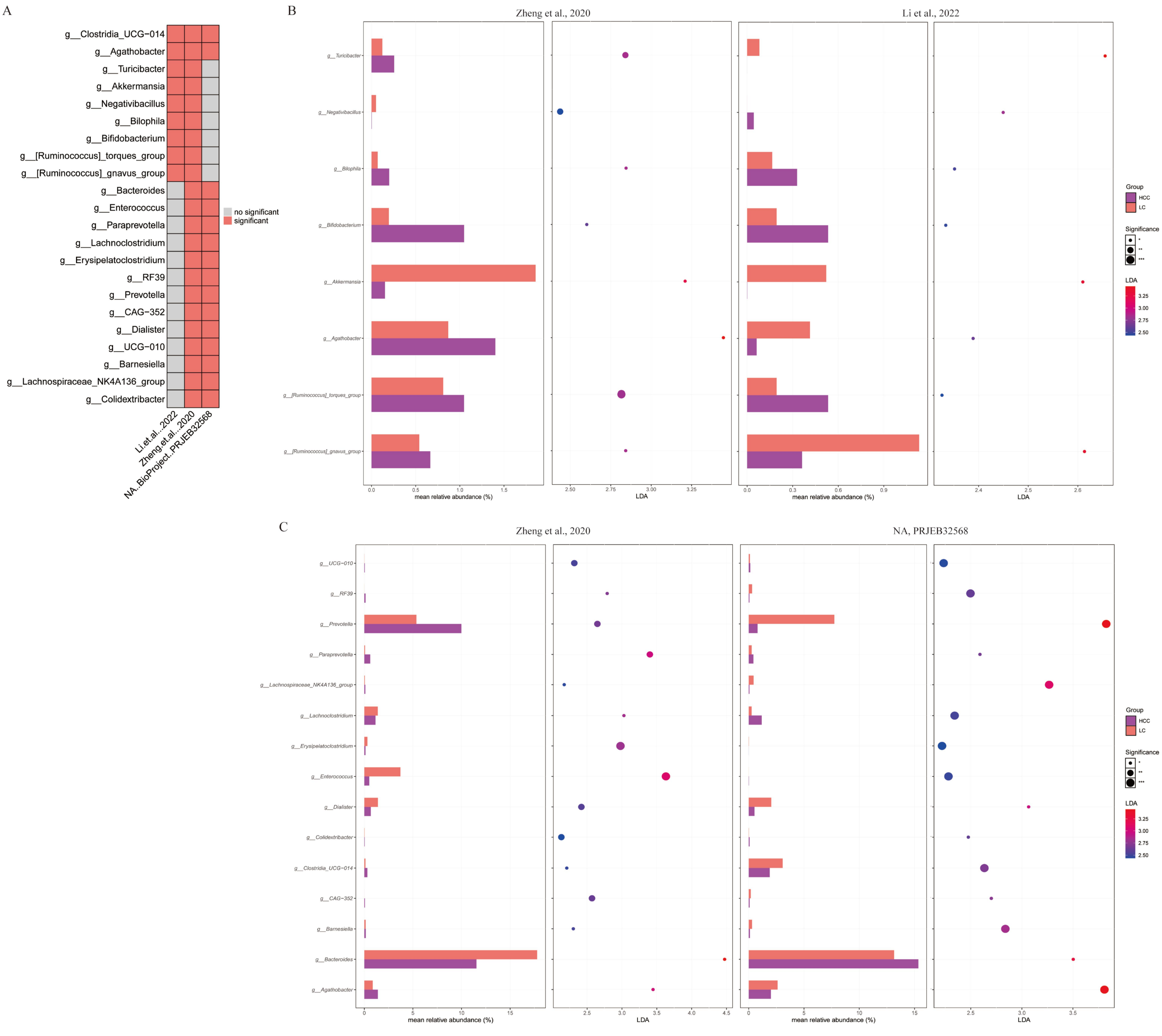

Figure S10 The integration of LEfSe-based methods (LDA score ≥ 2 and p < 0.05) identifies crucial genera that significantly increase in the occurrence of HCC. *HCC* Hepatocellular carcinoma, * *P*<0.05, ** *P*<0.01, *** *P*<0.001.

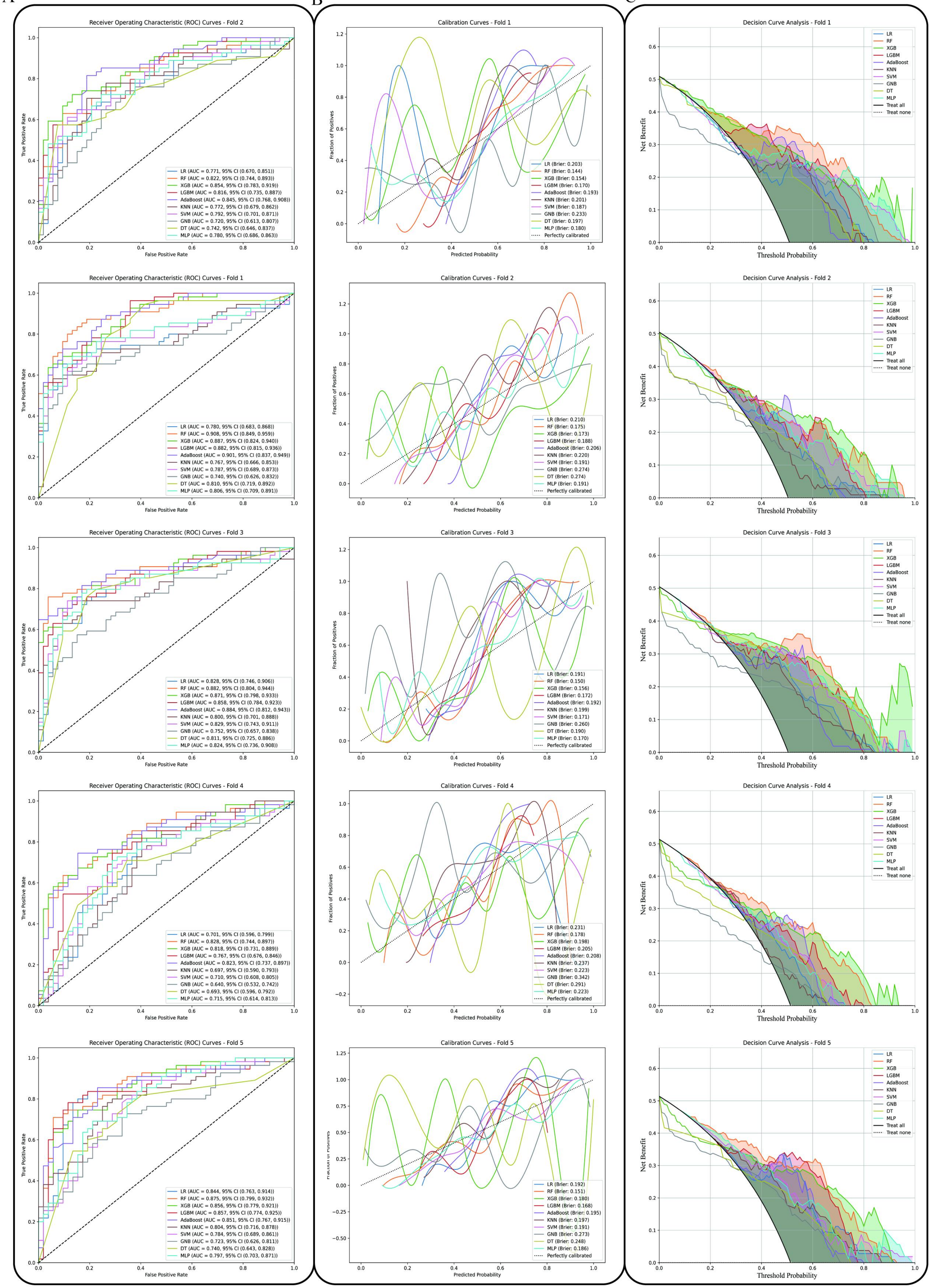

Figure S11 Evaluation of 10 machine learning models using five-fold cross-validation. [A] Receiver operating characteristic curves; [B] Calibration curves; [C] Decision curve analysis. In the five-fold cross-validation, the integrated dataset was randomly partitioned into five equal-sized folds. Each fold was held out once as the validation set while the remaining four folds were used for training, and this process was repeated five times.

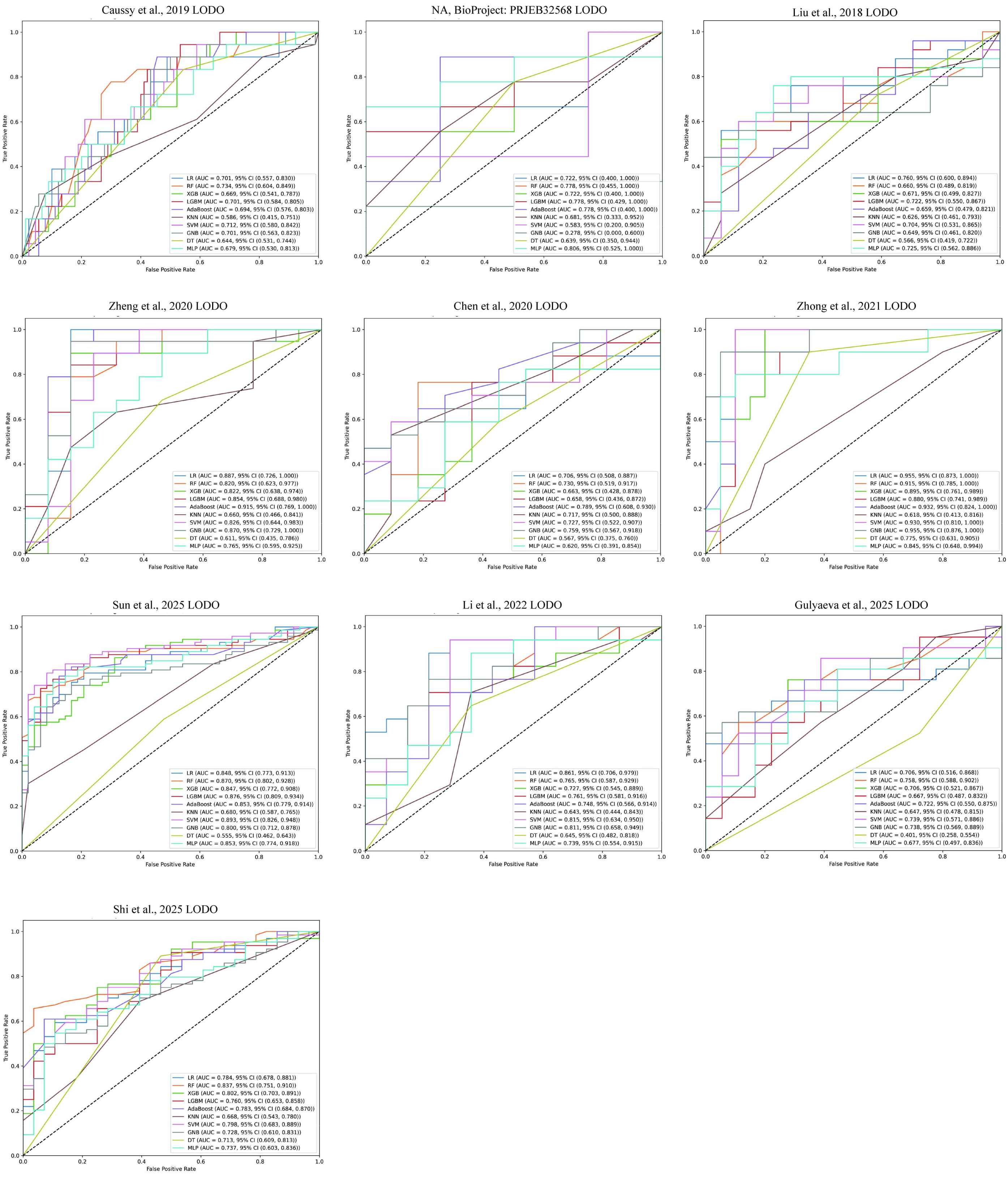

Figure S12 Receiver operating characteristic curves of 10 machine learning models in the LODO analysis. In the LODO analysis, all datasets except one were used for training, and the left-out dataset was then used for testing. *LODO* leave-one-dataset-out.

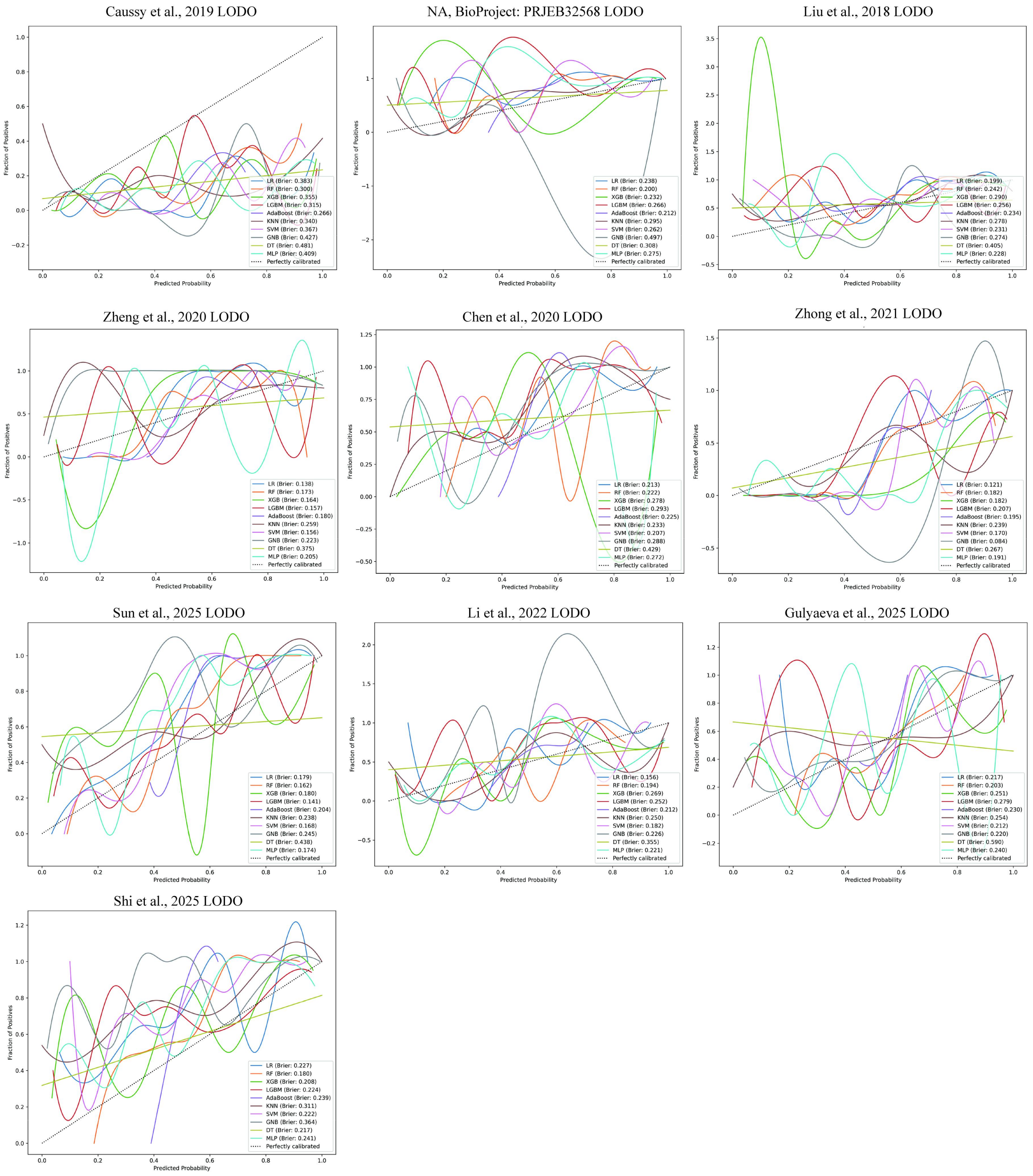

Figure S13 Calibration curves of 10 machine learning models in the LODO analysis. In the LODO analysis, all datasets except one were used for training, and the left-out dataset was then used for testing. *LODO* leave-one-dataset-out.

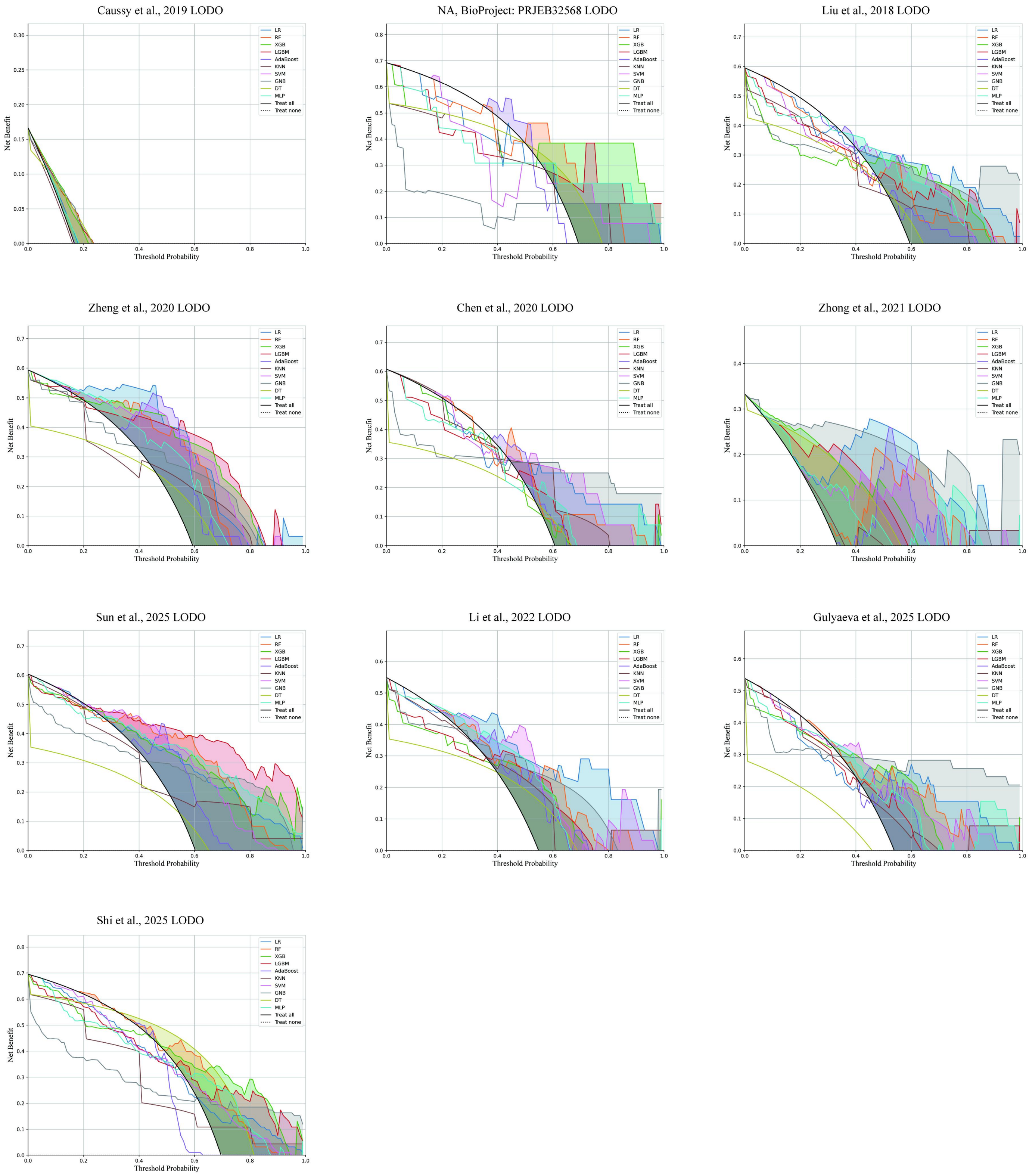

Figure S14 Decision curve analysis of 10 machine learning models in the LODO analysis. In the LODO analysis, all datasets except one were used for training, and the left-out dataset was then used for testing. *LODO* leave-one-dataset-out.

Table S1 Search Strategy

No.	Terms	Comments
#1	("Liver Cirrhosis"[MeSH Terms]) OR ("cirrhos*"[All Fields] OR "hepatic cirrhosis"[All Fields] OR "liver cirrhosis"[All Fields] OR "lc"[All Fields] OR "clc"[All Fields] OR "hepatic fibrosis"[All Fields])	liver cirrhosis
#2	(("gut"[Text Word] OR "intestinal*"[Text Word]) AND "microbiota"[MeSH Terms]) OR "microbe*"[Text Word] OR "microbiome*"[Text Word] OR "flora*"[Text Word] OR "microorganism*"[Text Word] OR "microflora*"[Text Word]	microbiota
#3	(#1 AND #2)	

Table S2 The reads were trimmed using the following parameters in the DADA2 plugin for each study

Study	--p-trim-left-f	--p-trunc-len-f	--p-trim-left-r	--p-trunc-len-r
Shi et al., 2025	0	249	0	247
Sun et al., 2025	0	240	0	230
Liu et al., 2018	5	250	5	250
Caussy et al., 2019*	--p-trim-left 5	--p-trunc-len 150		
NA, PRJEB32568*	--p-trim-left 0	--p-trunc-len 250		
Iebba et al., 2018	12	251	6	200
Zheng et al., 2020	6	250	6	250
Chen et al., 2020	0	244	0	213
Zhong et al., 2021	4	296	4	276
Li et al., 2022	0	235	0	235
Gulyaeva et al., 2025	0	154	0	153

Paired-end sequencing was used with the DADA2 "qiime dada2 denoise-paired" command in QIIME 2.
* Single read sequencing was used with the DADA2 " qiime dada2 denoise-single" command in QIIME 2 with parameters "--p-trim-left" and "--p-trunc-len".

Table S3 The R code for arcsine square root transformation and z-score normalization

```
# Load necessary packages
install.packages("microeco")
install.packages("magrittr")
install.packages("ape")
install.packages("dplyr")
install.packages("tidyr")

library(microeco)
library(magrittr)
library(ape)
library(dplyr)
library(tidyr)

# Import data files
otu_table <- read.delim('otu_table.tsv', row.names = 1, sep = '\t',
stringsAsFactors = FALSE, check.names = FALSE)
taxonomy <- read.delim('taxonomy.tsv', row.names = 1, check.names =
FALSE)
tree <- read.tree("rooted_tree.tre")

set.seed(123)  # Set random seed

taxonomy %<>% tidy_taxonomy  # Format the taxonomy table

# Create a microbiome dataset object (without group information)
dataset <- microtable$new(
  otu_table = otu_table,
  tax_table = taxonomy,
  phylo_tree = tree
)

# Data preprocessing
dataset$tidy_dataset()  # Unify data dimensions
dataset$filter_pollution(taxa = c("mitochondria", "chloroplast"))  #
Filter out mitochondrial and chloroplast sequences
dataset$tidy_dataset()  # Reorganize the data
print(dataset)  # Display basic dataset information

# Calculate taxonomic abundance
dataset$cal_abund()

# Extract the taxonomic abundance table
taxa_abund <- dataset$taxa_abund$Taxon
```

```r
# Perform arcsine square root transformation
taxa_abund_asin <- as.data.frame(asin(sqrt(taxa_abund)))

# Perform z-score standardization (standardize by taxon rows)
taxa_abund_z <- as.data.frame(t(scale(t(taxa_abund_asin))))

# Restore original dimensions and save results
colnames(taxa_abund_z) <- colnames(taxa_abund_asin)
rownames(taxa_abund_z) <- rownames(taxa_abund_asin)
dataset$taxa_abund$Taxon <- taxa_abund_z

# Export the transformed abundance table
write.csv(dataset$taxa_abund$Taxon,
        file = "taxa_abund_Taxon_transformed.csv",
        row.names = TRUE)
```

Table S4 Hyperparameter grid of 10 ML models.

Model	Hyperparameter grid
**LR**	class_weight = [{0: 1, 1: 1}, {0: 1, 1: 2}, {0: 1, 1: 3}, {0: 1, 1: 4}, {0: 1, 1: 5}]<br>param_grid = [<br>{<br>'penalty': ['l1'],<br>'C': C_values,<br>'solver': ['liblinear'],<br>'class_weight': class_weight<br>},<br>{<br>'penalty': ['l2'],<br>'C': C_values,<br>'solver': ['liblinear', 'lbfgs'],<br>'class_weight': class_weight<br>}<br>]
**RF**	param_grid = {<br>'n_estimators': [25, 50, 100],<br>'max_depth': [None, 5, 10, 15],<br>'min_samples_split': [2, 5, 8],<br>'min_samples_leaf': [1, 3, 5],<br>'max_features': ['sqrt', 'log2', None]<br>}
**XGB**	max_depths = [2, 3, 4, 5]<br>learning_rates = [0.01, 0.05, 0.1, 0.15, 0.2]<br>subsamples = [0.6, 0.7, 0.8, 0.9, 1.0]<br>colsample_bytrees = [0.3, 0.4, 0.5, 0.6, 0.7]<br>gammas = [0, 0.1, 0.2, 0.3, 0.4, 0.5]<br>lambdas = [0.01, 0.1, 0.5, 1.0, 1.5]<br>min_child_weights = [1, 2, 3, 4, 5]<br>param_grid = list(product(<br>max_depths,<br>learning_rates,<br>subsamples,<br>colsample_bytrees,<br>gammas,<br>lambdas,<br>min_child_weights<br>))<br>param_grid = random.sample(param_grid, 200)<br>results = []<br>for max_depth, learning_rate, subsample, colsample_bytree, gamma, lambd, min_child_weight in param_grid:<br>params = {<br>'max_depth': max_depth,<br>'learning_rate': learning_rate,<br>'subsample': subsample,<br>'colsample_bytree': colsample_bytree,<br>'gamma': gamma,<br>'lambda': lambd,<br>'objective': 'binary:logistic',<br>'eval_metric': 'auc' }

**LGBM**	param_grid = {<br>'num_leaves': [5, 10, 15, 20],<br>'max_depth': [3, 5, 7, 9],<br>'learning_rate': [0.01, 0.05, 0.1],<br>'n_estimators': [25, 50, 75],<br>'subsample': [0.3, 0.5, 0.7, 1.0],<br>'colsample_bytree': [0.3, 0.5, 0.7],<br>'reg_alpha': [0, 0.1, 0.3, 0.5],<br>'reg_lambda': [0, 0.1, 0.3, 0.5],<br>'min_data_in_leaf': [5, 10, 15, 20],<br>'min_sum_hessian_in_leaf': [1e-3, 1e-2],<br>'verbose': [-1]<br>}
**AdaBoost**	param_grid = {<br>'n_estimators': range(100, 300, 20),<br>'learning_rate': [0.01, 0.05, 0.1, 0.5, 1.0],<br>}
**KNN**	param_grid = {<br>'n_neighbors': list(range(5, 200)),<br>'weights': ['uniform', 'distance'],<br>'algorithm': ['auto', 'ball_tree', 'kd_tree', 'brute'],<br>'leaf_size': [1, 5, 7, 9, 12, 15],<br>'p': [1, 2]<br>}
**SVM**	param_grid = {<br>'C': np.linspace(0.1, 10, 10),<br>'gamma': np.linspace(0.1, 10, 10),<br>'kernel': ['linear', 'rbf']<br>}
**GNB**	param_grid = {<br>'var_smoothing': np.logspace(-12, -4, 150)
**DT**	param_grid = {<br>'criterion': ['gini', 'entropy'],<br>'max_depth': [None, 3, 5, 7, 10],<br>'min_samples_split': [2, 5, 6, 7, 8, 10],<br>'min_samples_leaf': [1, 2, 4, 5, 6, 7, 8]<br>}
**MLP**	param_grid = {<br>'hidden_layer_sizes': [(50,), (100,), (50, 50), (100, 50)],<br>'activation': ['relu', 'tanh'],<br>'solver': ['adam', 'sgd'],<br>'learning_rate_init': [0.0001, 0.001, 0.01, 0.1],<br>'max_iter': [1000, 2000]<br>}

Abbreviations: LR, Logistic Regression; RF, Random Forest; XGB, Extreme Gradient Boosting; LGBM, Light Gradient Boosting Machine; AdaBoost, Adaptive Boosting; SVM, Support Vector Machine; KNN, KNearest Neighbors; GNB, Gaussian Naïve Bayes; DT, Decision Tree; MLP, Multilayer Perceptron.

Table S5 Diagnostic methods and criteria for LC in the included studies

Author, Year	Definition of LC group	Definition of control group	Definition of HCC group	Exclusion criteria
Shi et al., 2025	based on liver biopsy, ultrasound, elastography, imaging, or endoscopy showing signs of portal hypertension.	Healthy Control	NA	age less than 18 or over 70 years; antibiotics, probiotics, proton pump inhibitors, and lactulose treatment within the last three months; hypertension, diabetes, or metabolic syndrome; liver tumors or other malignancies; other viral hepatitis, alcoholic hepatitis, autoimmune liver disease, or human immunodeficiency virus infection; and alcoholism, pregnancy, or incomplete information.
Sun et al., 2025	NA	Healthy Control	NA	alcoholic liver disease, autoimmune liver disease, fatty liver disease, chronic gastrointestinal disease, or other viral infections such as hepatitis C virus or human immunodeficiency virus; diabetes, obesity, or metabolic syndrome; use of antibiotics, probiotics, or proton pump inhibitors within one month; and pregnancy or lactation.
Gulyaeva et al., 2025	based on the Update to the Society of Radiologists in Ultrasound Liver Elastography Consensus Statement.	Healthy Control	NA	taking drugs that affect the gut microbiota (pro-, pre-, syn- or antibiotics) or alcohol for 6 weeks before inclusion in the study, the presence of gut diseases or other diseases that significantly affect the composition of the gut microbiota including solid tumors.
Li et al., 2022	based on iconography examination, positive pathological examinations, viral serologic testing, or chronic liver disease background.	Healthy Control	HCC: based on iconography examination, positive pathological examinations, viral serologic testing, or chronic liver disease background.	history of antibiotic, microecological preparation, or immunosuppressant treatment within the past 4 weeks; diagnosis of diabetes mellitus, autoimmune disease, such as multiple sclerosis, rheumatoid arthritis, hypertension, coronary heart disease, or metabolic syndrome; and pregnancy or lactation. Written Informed consent was obtained from all participants.
Zhong et al., 2021	long-term alcohol consumption history or drink more than 40 g per day; the patients were not suffering from viral hepatitis, drug hepatitis, autoimmune liver disease or other specific etiology of liver disease; clinical imageological examination (ultrasonic B or CT)	Healthy Control	NA	if the patients were suffering from viral hepatitis, drug hepatitis, autoimmune liver disease or other specific etiology of liver disease, if the patients were suffering from the diseases that confirmed related to microbiome, such as inflammatory bowel disease, gastrointestinal tumors, heart disease, diabetes and so on, if the patients regularly use or recently use specific drugs, which may affect the condition of gut microbiota, such as proton pump inhibitor (PPI), laxatives and so on.

Zheng et al., 2020	using Magnetic Resonance (MR), Computed Tomography (CT), HE staining of pathological sections, serum AFP levels, and chronic liver disease history.	Healthy Control	HCC: using Magnetic Resonance (MR), Computed Tomography (CT), HE staining of pathological sections, serum AFP levels, and chronic liver disease history.	all participants had not received prior anticancer treatment; no other diseases, such as heart disease or hypertension, were present; and they did not take drugs such as antibiotics, prebiotics, or other drugs in the last 6 months. In addition, healthy participants who had intestinal and liver-related diseases were also excluded.
Chen et al., 2020	computed tomography (CT) alone, magnetic resonance imaging (MRI) alone, ultrasonography alone, CT plus MRI, CT plus ultrasonography, MRI plus ultrasonography, CT plus MRI plus ultrasonography. other causes of liver disease such as NAFLD, autoimmune liver disease, hepatitis A, hepatitis C, hepatitis D, hepatitis E and liver parasite infection were excluded.	Healthy Control	NA	symptoms of digestive system disorders, such as hematochezia, constipation, abdominal distention, abdominal pain, diarrhea, and jaundice within 1 month; abnormal results of several tests, including: routine blood, liver function, renal function, blood fat, fasting blood glucose, HBsAg, routine fecal and fecal occult blood tests; an enteritis diagnosis within 1 month; chronic obstructive pulmonary disease, renal insufficiency and other systemic diseases; autoimmune disease; chronic fatigue syndrome and neuropsychic disease; a history of antibiotic, microecological preparation, gastrointestinal motility medicine, laxative, weight loss drug, glucose lowering, blood fat regulation, glucocorticoid, or immunosuppressant treatment within 1 month; history of organic diseases in the digestive system, such as gastrointestinal polyposis, ulcers, cirrhosis, and malignancies; history of gastrointestinal surgery; or a family history of diabetes, hypertension, coronary heart disease, metabolic syndrome, etc.

Caussy, et al. 2019	MRE threshold ≥ 3.63 kPa (based on American Association for the Study of Liver Study Practice Guidelines)	Healthy Control	NA	regular and excessive alcohol consumption within 2 years of recruitment (≥14 drinks/week for men or ≥7 drinks/week for women); use of hepatotoxic drugs or drugs known to cause hepatic steatosis; evidence of liver diseases other than NAFLD, including viral hepatitis (detected with positive serum hepatitis B surface antigen or hepatitis C viral RNA), Wilson's disease, hemochromatosis, alpha-1 antitrypsin deficiency, autoimmune hepatitis, and cholestatic or vascular liver disease; clinical or laboratory evidence of chronic illnesses associated with hepatic steatosis, including human immunodeficiency virus infection (HIV), celiac disease, cystic fibrosis, lipodystrophy, dysbetalipoproteinemia, and glycogen storage diseases; evidence of active substance abuse, significant systemic illnesses, contraindication(s) to MRI, pregnant or trying to become pregnant, or any other condition which, in the investigator's opinion, may affect the patient's competence or compliance in completing the study.
Liu et al., 2018	based on the Diagnosis of cirrhosis and portal hypertension: imaging, non-invasive markers of fibrosis and liver biopsy	Healthy Control	NA	presence of malignancy, infections, known GI or renal disease or significant respiratory or cardiac dysfunction; diagnosis of diabetes mellitus, untreated thyroid dysfunction or previous gastrointestinal surgery; history of an autoimmune disease such as multiple sclerosis, rheumatoid arthritis, IBS and IBD.
Iebba et al., 2018	proven through liver biopsy or based on clinical, biochemical and ultrasonographic signs	Healthy Control	NA	diagnosis of infection (based on fever, leukocytosis, elevated C Reactive protein (CRP), erythrosedimentation rate (ESR), procalcitonin, clinical symptoms, and positive microbiological cultures when present), use of systemic antibiotics in the last 3 months, variceal bleeding within the last 4 weeks, or alcohol or illicit drug intake within the last 3 months. Lactulose or rifaximin therapy was not considered cause for exclusion. No patient took other drugs that could potentially affect the microbiota (such as metformin). Patients with any type of immunodeficiency (HIV, immunosuppression) or with a diagnosis of hepatocellular carcinoma without Milano criteria were excluded.
NA, PRJEB32568	NA	NA	NA	NA

NA Not Applicable, *LC* liver cirrhosis, *HCC* Hepatocellular carcinoma.
